# Real-World Treatment Patterns and Clinical Outcomes After First-Line Therapy in Patients with KRAS G12C-Mutant Advanced Non-Small-Cell Lung Cancer in the United States

**DOI:** 10.3390/cancers18172869

**Published:** 2026-09-04

**Authors:** Kristin M. Sheffield, Tarun Puri, Kelli Thoele, Himanshu Karu, Arti Mansharamani, Dipesh Uprety

**Affiliations:** 1 Eli Lilly and Company, Indianapolis, IN 46285, USA; puri_tarun@lilly.com (T.P.); thoele_kelli@lilly.com (K.T.); himanshukaru@hotmail.com (H.K.); mansharamani24@gmail.com (A.M.); 2 Marlene and Stewart Greenebaum Comprehensive Cancer Center, University of Maryland School of Medicine, Baltimore, MD 21201, USA; upretydipesh@gmail.com

**Keywords:** non-small-cell lung cancer, *KRAS* G12C, real-world outcomes, patient characteristics, unmet need

## Abstract

Background: Approximately 13% of NSCLC cases have *KRAS* G12C mutations. As therapeutic strategies targeting *KRAS* G12C-mutant NSCLC evolve, it is important to understand clinical presentation and current outcomes for these patients. Methods: This retrospective study used data from two US nationwide databases, an electronic health records (EHR) database and a clinico-genomic database (CGDB) of EHR data linked to data from comprehensive genomic profiling tests. Eligible patients had advanced NSCLC, initiated first-line therapy from August 2018 to December 2022, and had *KRAS* test results. Clinicopathologic characteristics, treatments, real-world progression-free survival (rwPFS), and overall survival (OS) were analyzed. Results: There were 1227 patients with *KRAS* G12C-mutant NSCLC in the EHR database and 447 in the CGDB. First-line regimen was platinum-based chemotherapy plus pembrolizumab for 46% and pembrolizumab monotherapy for 20%. Less than 40% of patients received second-line therapy. Median (95% CI) OS for *KRAS* G12C-mutant NSCLC patients in the EHR was 17.0 (15.2–18.9) months. Variables significantly associated with shorter OS included PD-L1 <1%, brain metastases, STK11 co-mutation, and poor performance status. Patients treated with platinum-based chemotherapy plus pembrolizumab had median rwPFS of 5.3 (4.5–7.3) months and OS of 12.8 (11.1–17.3) months in the CGDB; median OS was 15.6 (12.5–18.6) months in the EHR. Patients with PD-L1 ≥ 50% treated with pembrolizumab monotherapy had median rwPFS of 4.6 (3.0–15.6) months and OS of 20.4 (10.3–38.5) months in the CGDB; median OS was 22.1 (18.7–30.7) in the EHR. Conclusions: These data provide a real-world benchmark of outcomes for patients with *KRAS* G12C-mutant NSCLC receiving the current standard of care and indicate an unmet need for more effective first-line therapies.

## Simple Summary

Around 13% of patients diagnosed with non-small-cell lung cancer (NSCLC) have tumors with KRAS G12C mutations. This study used data from electronic health records databases to describe the characteristics of these patients, the initial treatments they received, and their survival following treatment with chemotherapy plus immunotherapy and immunotherapy alone. Two-thirds of patients received chemotherapy plus immunotherapy (46%) or immunotherapy alone (20%) as initial treatment. Less than 40% of patients received subsequent second-line treatment for their lung cancer. Median overall survival was 13–16 months for patients treated with chemotherapy plus immunotherapy and 20–22 months for patients treated with immunotherapy alone whose tumors had high PD-L1 expression. These results demonstrate real-world outcomes for patients with KRAS G12C-mutant NSCLC in clinical practice, providing a benchmark for outcomes on the current standard of care and highlighting the significant unmet need for more effective first-line treatment options.

## 1. Introduction

Kirsten rat sarcoma viral oncogene homolog (*KRAS*) mutations occur in approximately 35% of non-squamous non-small-cell lung cancer (NSCLC) cases [1]. *KRAS* G12C is the most common variant, accounting for approximately 37–40% of *KRAS* mutations and 9–14% of NSCLC cases in US genomic database studies [1,2,3]. *KRAS* G12C mutations are associated with smoking history and are often found in older populations with comorbid conditions [4,5,6,7].

Current first-line treatment approaches for patients with advanced or metastatic *KRAS* G12C-mutant NSCLC are not directed specifically at the *KRAS* G12C mutation. Standard first-line treatment as per clinical practice guidelines includes an immune checkpoint inhibitor (ICI) alone or in combination with platinum-based doublet chemotherapy [8,9,10]. *KRAS* G12C mutations are associated with elevated tumor mutational burden (TMB) and higher Programmed Cell Death Ligand 1 (PD-L1) expression compared with NSCLC tumors with other oncogenic drivers or *KRAS* wild-type tumors [1,11,12]. As a result, *KRAS* G12C-mutant NSCLC is sensitive to PD-1/PD-L1 inhibition, unlike lung cancers with other oncogenic driver mutations, such as epidermal growth factor receptor (*EGFR)*, anaplastic lymphoma kinase *(ALK)*, or rearranged during transfection *(RET)* [13,14,15,16]. Two KRAS G12C inhibitors, sotorasib and adagrasib, have received accelerated approval from the FDA for patients with *KRAS* G12C-mutant NSCLC who have received at least one prior therapy [17,18]. Confirmatory Phase 3 trials demonstrated improvements in progression-free survival compared to docetaxel: 5.6 months vs. 4.5 months for sotorasib (hazard ratio [HR] = 0.66, 95% confidence interval [CI] = 0.51–0.86) and 5.5 months vs. 3.8 months for adagrasib (HR = 58, 95% CI = 0.45–0.76) [19,20]. Multiple next-generation KRAS G12C inhibitors are currently being evaluated in clinical trials. Preclinical data demonstrating additive or synergistic effects of KRAS G12C inhibitors combined with immunotherapy, as well as preliminary efficacy and safety data of these combinations in 1L NSCLC, have generated interest in exploring combination therapies to enhance efficacy and overcome resistance mechanisms [21,22,23,24,25,26,27,28]. There are ongoing Phase 3 clinical trials investigating KRAS G12C inhibitors in the first-line setting in combination with immunotherapy, with or without platinum-based chemotherapy, and in combination with platinum-based chemotherapy [29,30,31,32,33,34].

As therapeutic strategies targeting *KRAS* G12C-mutant NSCLC continue to evolve, it is important to understand real-world outcomes for patients receiving the standard-of-care treatments incorporated in ongoing Phase 3 trials. Real-world outcomes for patients with *KRAS* G12C-mutant NSCLC treated with first-line chemoimmunotherapy and immunotherapy in clinical practice have not been well characterized, with previous studies being limited by small sample sizes, not reporting outcomes separately by regimen, or using older data collected before the introduction of KRAS G12C inhibitors in the second-line setting. Hence, there is a need for updated real-world data on patients with *KRAS* G12C-mutant NSCLC to clearly understand the unmet needs and treatment gaps. This study used two large nationwide electronic health records databases to describe patient and tumor characteristics and treatment patterns for patients with *KRAS* G12C-mutant NSCLC and to evaluate outcomes following first-line treatment with chemoimmunotherapy and immunotherapy in the United States.

## 2. Materials and Methods

### 2.1. Data Sources and Patient Selection

This retrospective observational cohort study utilized two US-based datasets: the Flatiron Health-Foundation Medicine NSCLC Clinico-Genomic Database (FH-FMI CGDB) and the electronic health record-derived deidentified Flatiron Health Research Database (hereafter, EHR database) [35]. These datasets comprise deidentified patient-level structured and unstructured data, curated through technology-enabled abstraction. The data originate from approximately 280 cancer clinics (~800 sites of care) in the United States. In the FH-FMI CGDB, clinical data from the Flatiron Health Research Database are linked to genomic data derived from FMI’s comprehensive genomic profiling tests by deterministic matching [35]. Patients in these datasets were diagnosed with lung cancer, had at least two documented clinical visits occurring on or after 1 January 2011, pathology consistent with NSCLC, and were diagnosed with Stage IIIB-IV NSCLC or early-stage NSCLC with subsequent recurrent or progressive disease.

Additional eligibility criteria were applied for inclusion in this study. Patients received first-line therapy from 1 August 2018 to 31 December 2022 and received *KRAS* test results (next-generation sequencing for FH-FMI CGDB) <90 days before and <30 days after the start of first-line therapy. Data were available through 30 September 2023 for the FH-FMI CGDB and 30 November 2023 for the broader EHR data. Patients were excluded if they had a regimen with a “Clinical Study Drug” in their first-line or second-line treatment, or if they died within 30 days from the start date of first-line therapy.

### 2.2. Variables

Patient characteristics included age, gender, race, smoking status, and Eastern Cooperative Oncology Group (ECOG) performance status (PS) score. Disease characteristics included histology, stage at initial diagnosis, sites of metastases, PD-L1 testing status and expression level, and biomarker status (KRAS, EGFR, ALK, ROS1, BRAF, MET exon 14 skipping, RET rearrangement, NTRK rearrangement, STK11, KEAP1, TP53).

First-line and second-line treatment patterns were reported based on oncology expert-defined, rules-based line of therapy. Primary endpoints for evaluating patient outcomes were real-world progression-free survival (PFS) and overall survival. Progression data were available in the FH-FMI CGDB only. PFS was measured from initiation of first-line treatment until the date of progression or the date of death in the absence of progression. Patients without an event were censored at their last clinic note date. Overall survival (OS) was evaluated from initiation of first-line treatment until the date of death. The 15th of the month was assigned as the death date. Patients without an event were censored at the date of the last confirmed activity.

### 2.3. Statistical Analysis

All analyses were conducted separately for the EHR database and the FH-FMI CGDB. Patient demographics, clinical characteristics, tumor characteristics, and treatment regimens were summarized and reported using descriptive statistics for the 1227 patients with *KRAS* G12C-mutant NSCLC in the EHR database and the 447 patients with *KRAS* G12C-mutant NSCLC in the CGDB (see Table S1 for attrition table for these cohorts). The Kaplan–Meier method [36] was used to evaluate OS for patients with KRAS G12C-mutant NSCLC in the EHR database by PD-L1 expression, presence of brain metastases, *STK11* mutation status, and *KEAP1* mutation status. Median OS estimates and 95% confidence intervals were reported. Multivariable Cox proportional hazards regression models [37] were used to evaluate demographic and clinical characteristics associated with OS. The EHR database was used for these analyses due to the anticipated larger sample size, which would enable evaluation of OS by PD-L1 expression, brain metastases, and co-mutation status, and the broader patient population (as the CGDB contains patients with Foundation Medicine comprehensive testing only).

We evaluated PFS and OS for patients with KRAS G12C-mutant NSCLC treated with platinum-based chemotherapy plus pembrolizumab and pembrolizumab monotherapy. Patients without a KRAS G12C mutation who received the regimens of interest were included as comparison groups for the PFS and OS analyses and subdivided into other *KRAS* mutation, *KRAS* wild type with driver mutation, and *KRAS* wild type without driver mutation (see Table S2 for the counts in each cohort). The Kaplan–Meier method was used to estimate PFS and OS for each subgroup. Median estimates and 95% confidence intervals were reported. Multivariable Cox proportional hazards regression models were used to estimate hazard ratios and 95% confidence intervals for the subgroups compared with patients with KRAS G12C-mutant NSCLC, adjusting for age, gender, race, histology, performance status, stage at initial diagnosis, smoking status, PD-L1 expression, brain metastases, *STK11* mutation, *KEAP1* mutation, and *TP53* mutation. Weighted Cox regression models were used for models with violations of the proportional hazards assumption. Analyses of PFS were conducted only in the CGDB because progression data were not available in the EHR database.

All statistical tests were conducted with a significance level defined as alpha < 0.05. Data analyses were performed using SAS Version 8.2 and R, Version 4.4.1. Missing data for variables are reported in the tables as a separate category. No imputation was conducted.

## 3. Results

### 3.1. Patient and Disease Characteristics

There were 1227 patients with *KRAS* G12C-mutant NSCLC who met the study criteria in the EHR database and 447 patients in the CGDB. The demographic and baseline clinical characteristics of the study population are given in Table 1. Median age was 69 years in the EHR and 70 years in the CGDB databases. Most patients had advanced-stage disease at diagnosis (80.7% and 76.1%), and nearly all had a history of smoking (98.3% and 98.7%) and non-squamous cell carcinoma histology (92.3% and 92%). About 25% of patients had documented brain metastases at the start of 1L treatment. Among patients in the EHR database, 31.9% had documented brain metastases at any time during follow-up. Approximately 19% of patients had performance status scores of ≥2.

Nearly half of patients with *KRAS* G12C-mutant NSCLC had tumors with PD-L1 ≥ 50% (44.7% and 45.3% of tested patients in the EHR and CGDB databases, respectively). In the EHR database, 22.2% of tested patients had tumors with STK11 mutations, and 12.1% had tumors with KEAP1 mutations. In the CGDB, 27.3% and 19.5% had tumors with STK11 and KEAP1 mutations, respectively. The prevalence of these co-mutations was significantly higher among patients with tumors with PD-L1 < 50% compared with patients with tumors with PD-L1 ≥ 50% (Table S4 and Table S5). In the EHR database, 34.1% of patients had tumors with STK11 mutations in the PD-L1 < 50% subgroup versus 7.4% in the PD-L1 ≥ 50% subgroup (*p* < 0.0001), and 16.2% of patients in the PD-L1 < 50% subgroup had tumors with KEAP1 mutations compared with 8.0% in the PD-L1 ≥ 50% subgroup (*p* = 0.0133). In the CGDB, 40.4% of patients in the PD-L1 < 50% subgroup had tumors with STK11 mutations compared with 12.8% in the PD-L1 ≥ 50% subgroup (*p* < 0.0001), and 26.5% of patients with PD-L1 < 50% had KEAP1 mutations compared with 12.0% of patients with tumors with PD-L1 ≥ 50% (*p* = 0.0044).

### 3.2. Treatment Patterns

Table 2 shows the top first-line and second-line regimens in patients with *KRAS* G12C-mutant NSCLC overall and the top first-line regimens for the subgroup with PD-L1 ≥ 50%. Across all PD-L1 levels, approximately 46% of patients received platinum-based chemotherapy with pembrolizumab and 20% received pembrolizumab monotherapy in each cohort. Among the subgroup of patients with PD-L1 ≥ 50%, pembrolizumab monotherapy was the top first-line regimen, with 43.4% of EHR patients and 36.8% of CGDB patients receiving it. For both cohorts, fewer than 40% of patients treated with first-line therapy received subsequent treatment (483/1227 patients [39.4%] in the EHR and 167/447 patients [37.4%] in the CGDB). Almost 30% of patients who received second-line treatment received KRAS G12C inhibitors, making it the most commonly used therapy.

Table 3 shows the demographic and clinical characteristics of patients with *KRAS* G12C-mutant NSCLC treated with platinum-based chemotherapy plus pembrolizumab, compared with those treated with pembrolizumab monotherapy, based on data from the EHR database. Patients treated with pembrolizumab monotherapy were more likely to be 75 years or older (46.8% vs. 24.3%), female (63.2% vs. 55.5%), and have performance status scores of ≥2 (27.6% vs. 17.3%) compared with patients treated with platinum-based chemotherapy plus pembrolizumab. The frequency of bone metastases was higher among patients treated with platinum-based chemotherapy plus pembrolizumab compared with patients treated with pembrolizumab monotherapy (48.0% vs. 34.8%, *p* = 0.0005); no other differences in sites of metastases were observed. Patients treated with pembrolizumab monotherapy were more likely to have tumors with PD-L1 ≥ 50% (81.6% vs. 31.1%), with 43% of pembrolizumab-treated patients having tumors with PD-L1 ≥ 90%. (Table S3 presents the detailed breakdown of PD-L1 staining percentage for each group.) Patients treated with pembrolizumab monotherapy were less likely to have tumors with STK11 co-mutations. Table S6 presents demographic and clinical characteristics for each treatment group among patients with tumors expressing PD-L1 ≥ 50%. There were no differences in the prevalence of STK11 or KEAP1 between groups.

### 3.3. Overall Survival Among KRAS G12C-Mutant NSCLC Patients (EHR Database)

**Figure 1:**
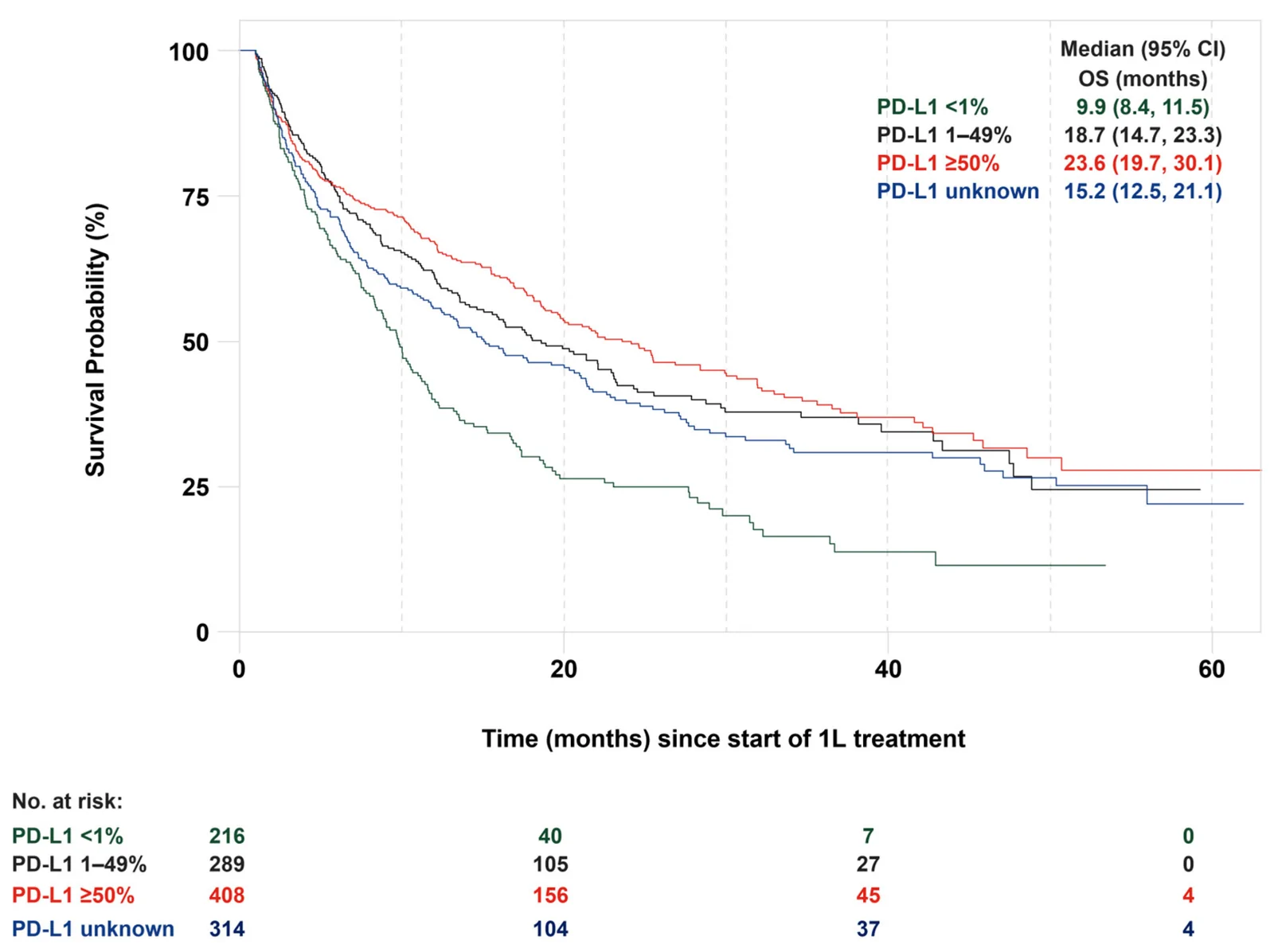
Overall survival for patients with *KRAS* G12C-mutant NSCLC by PD-L1 expression. Abbreviations: 1L, first-line; CI, confidence interval; *KRAS*, Kirsten rat sarcoma viral oncogene homolog; NSCLC, non-small-cell lung cancer; OS, overall survival; PD-L1, Programmed Cell Death Ligand 1.

**Figure 2:**
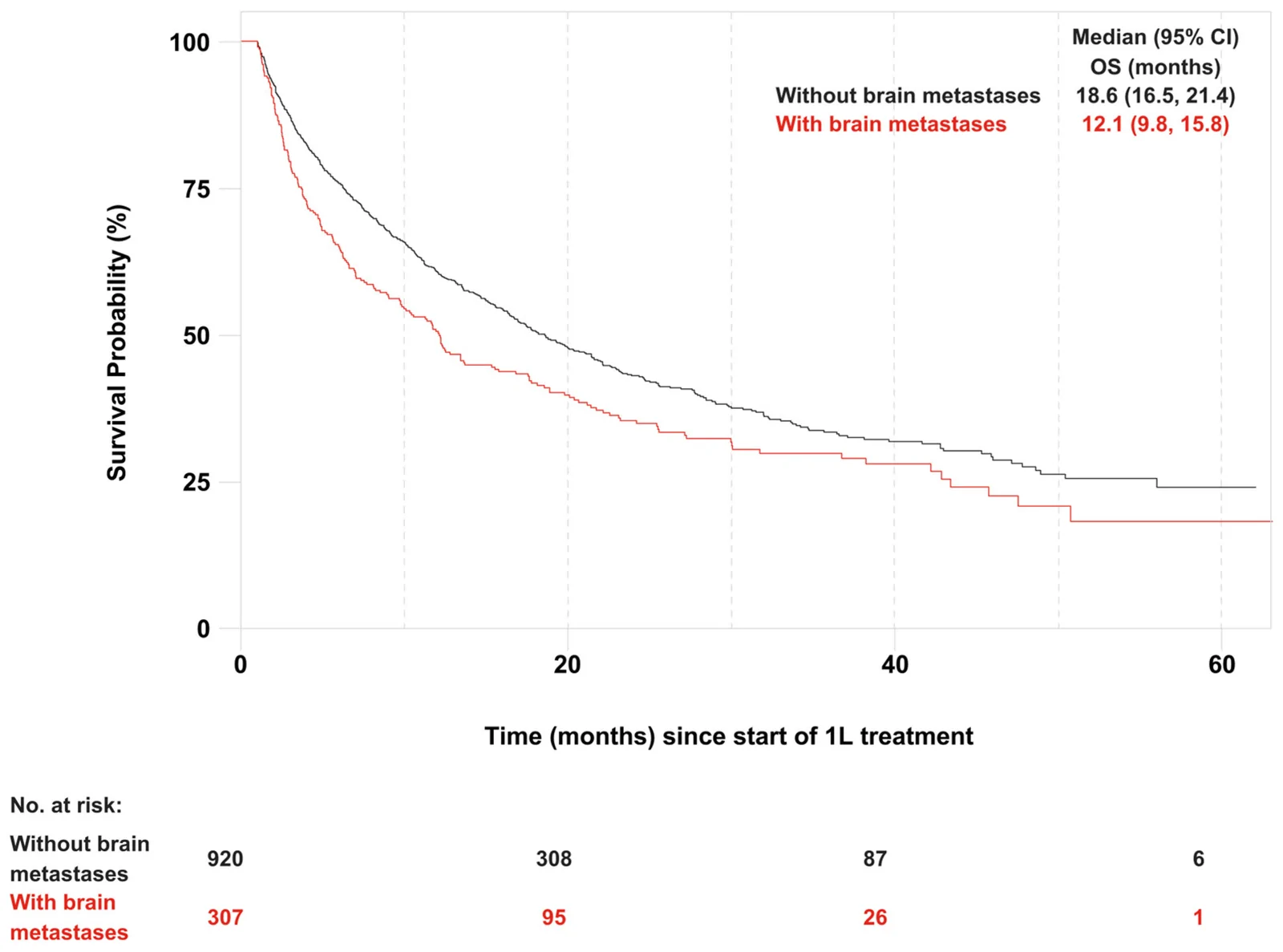
Overall survival for patients with *KRAS* G12C-mutant NSCLC by documented brain metastases at time of first-line therapy. Abbreviations: 1L, first-line; CI, confidence interval; *KRAS*, Kirsten rat sarcoma viral oncogene homolog; NSCLC, non-small-cell lung cancer; OS, overall survival.

**Figure 3:**
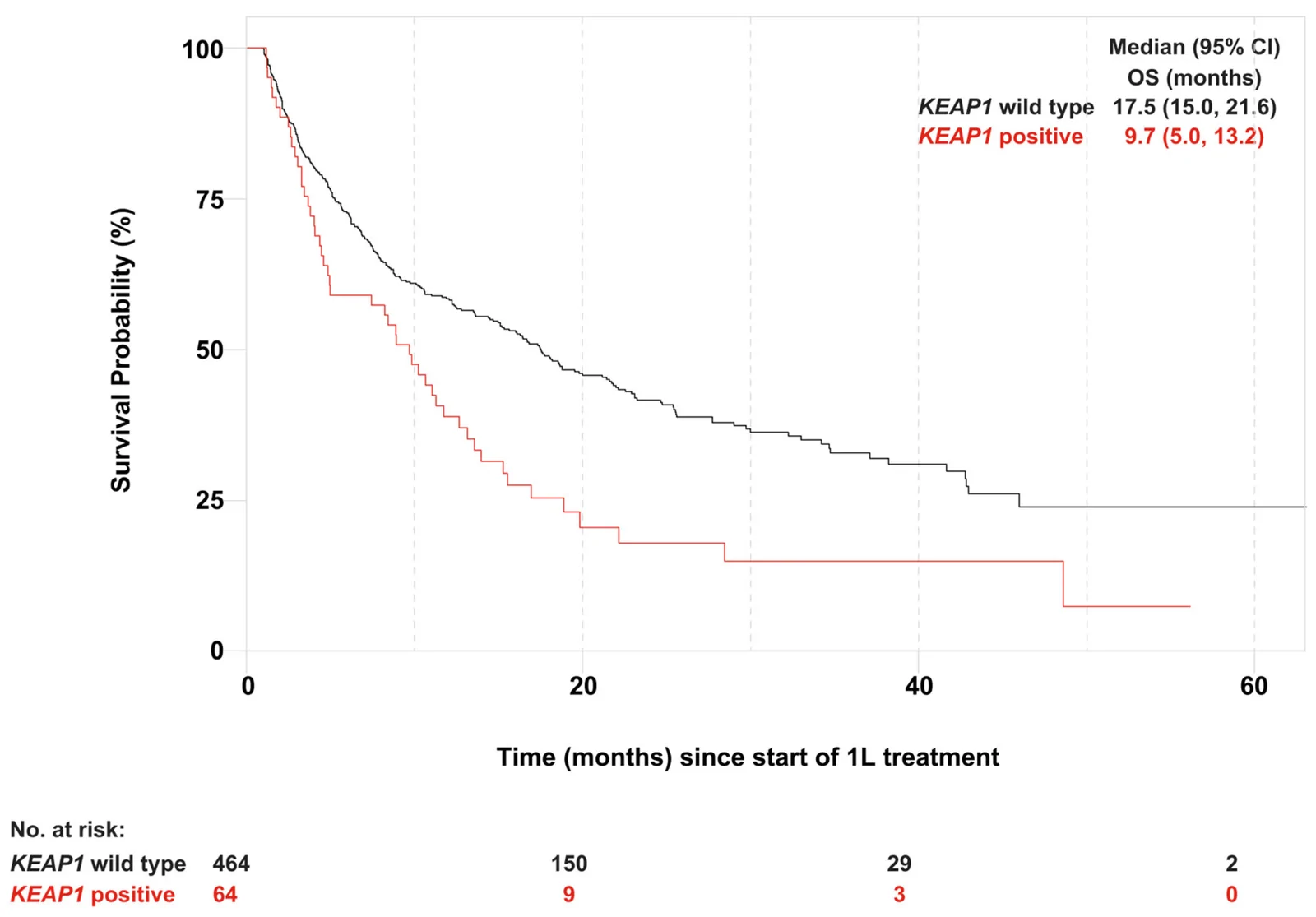
Overall survival for patients with *KRAS* G12C-mutant NSCLC by KEAP1 mutation status. Abbreviations: 1L, first-line; CI, confidence interval; KEAP1, Kelch-like ECH-associated protein 1; *KRAS*, Kirsten rat sarcoma viral oncogene homolog; NSCLC, non-small-cell lung cancer; OS, overall survival.

**Figure 4:**
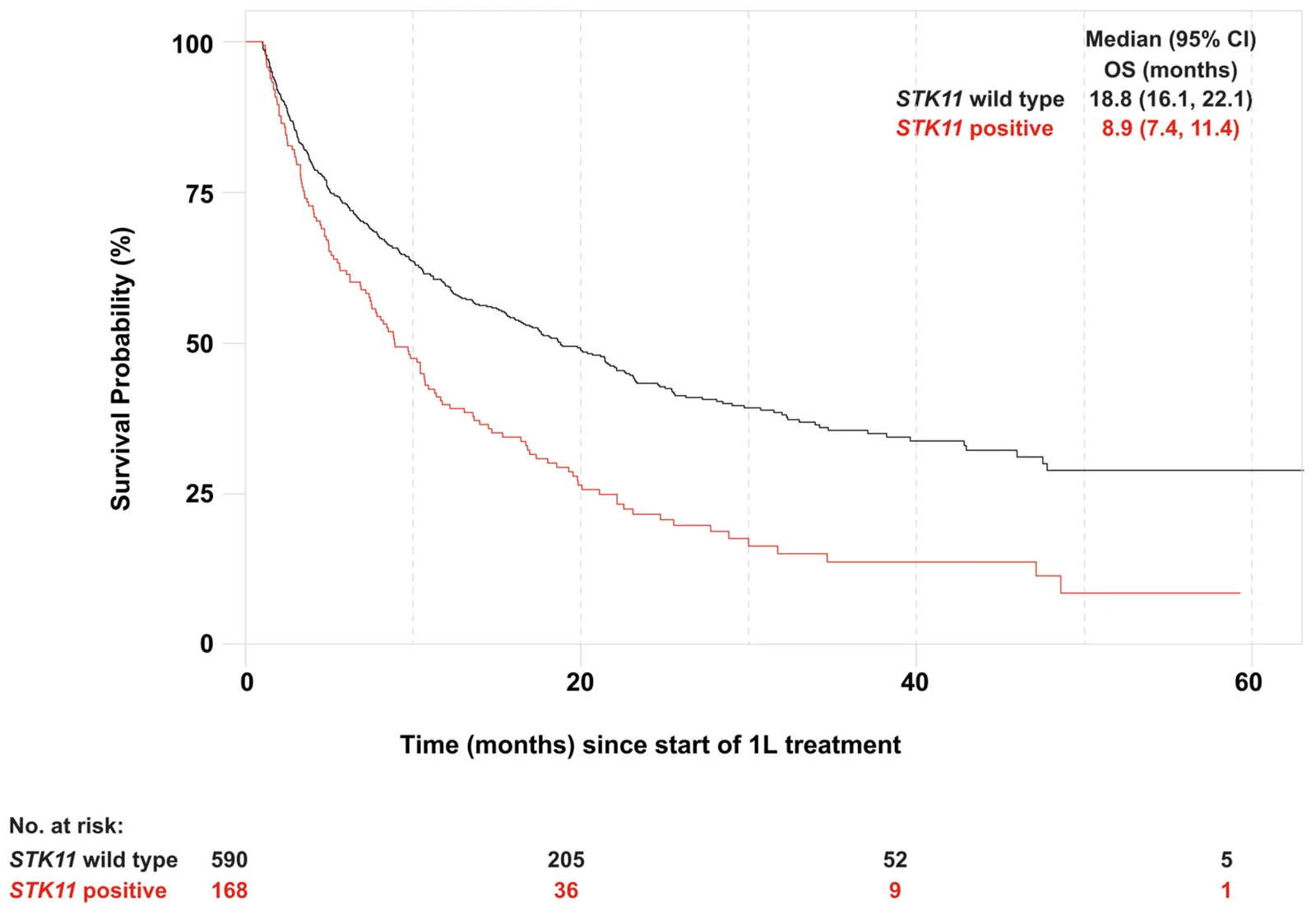
Overall survival for patients with *KRAS* G12C-mutant NSCLC by STK11 mutation status. Abbreviations: 1L, first-line; CI, confidence interval; *KRAS*, Kirsten rat sarcoma viral oncogene homolog; NSCLC, non-small-cell lung cancer; OS, overall survival; STK11, serine/threonine kinase 11.

Overall survival from the start of first-line treatment was evaluated among 1227 patients with *KRAS* G12C-mutant NSCLC in the EHR database, overall and by PD-L1 status, brain metastases at the start of first-line, STK11 mutation, and KEAP1 mutation. Median OS was 17.0 months (95% confidence interval [CI]: 15.2, 18.9) for the overall group. Figure 1 shows OS by PD-L1 status. Median OS was 9.9 (95% CI: 8.4, 11.5) months for patients with tumors with PD-L1 < 1%, 18.7 (95% CI: 14.7, 23.3) months for patients with tumors with PD-L1 1–49%, and 23.6 (95% CI: 19.7, 30.1) months for patients with tumors with PD-L1 ≥ 50% (Figure 1). Similar OS results by PD-L1 expression were observed among the subset of patients treated with platinum-based pembrolizumab plus chemotherapy (Figure S1). Median OS was 12.1 (95% CI: 9.8, 15.8) months for patients with documented brain metastases at the start of first-line therapy and 18.6 (95% CI: 16.5, 21.4) months for patients without documented brain metastases at the start of first-line therapy (Figure 2). Among patients with KEAP1 test results (n = 528), median OS was 9.7 (95% CI: 5.0, 13.2) months for patients with KEAP1 mutant NSCLC and 17.5 (95% CI: 15.0, 21.6) months for patients with KEAP1 wild-type NSCLC (Figure 3). Among patients with STK11 test results (n = 758), median OS was 8.9 (95% CI: 7.4, 11.4) months for patients with STK11 mutant NSCLC and 18.8 (95% CI: 16.1, 22.1) months for patients with STK11 wild-type NSCLC (Figure 4).

### 3.4. Predictors of OS Among Patients with KRAS G12C-Mutant NSCLC (EHR Database)

**Figure 5:**
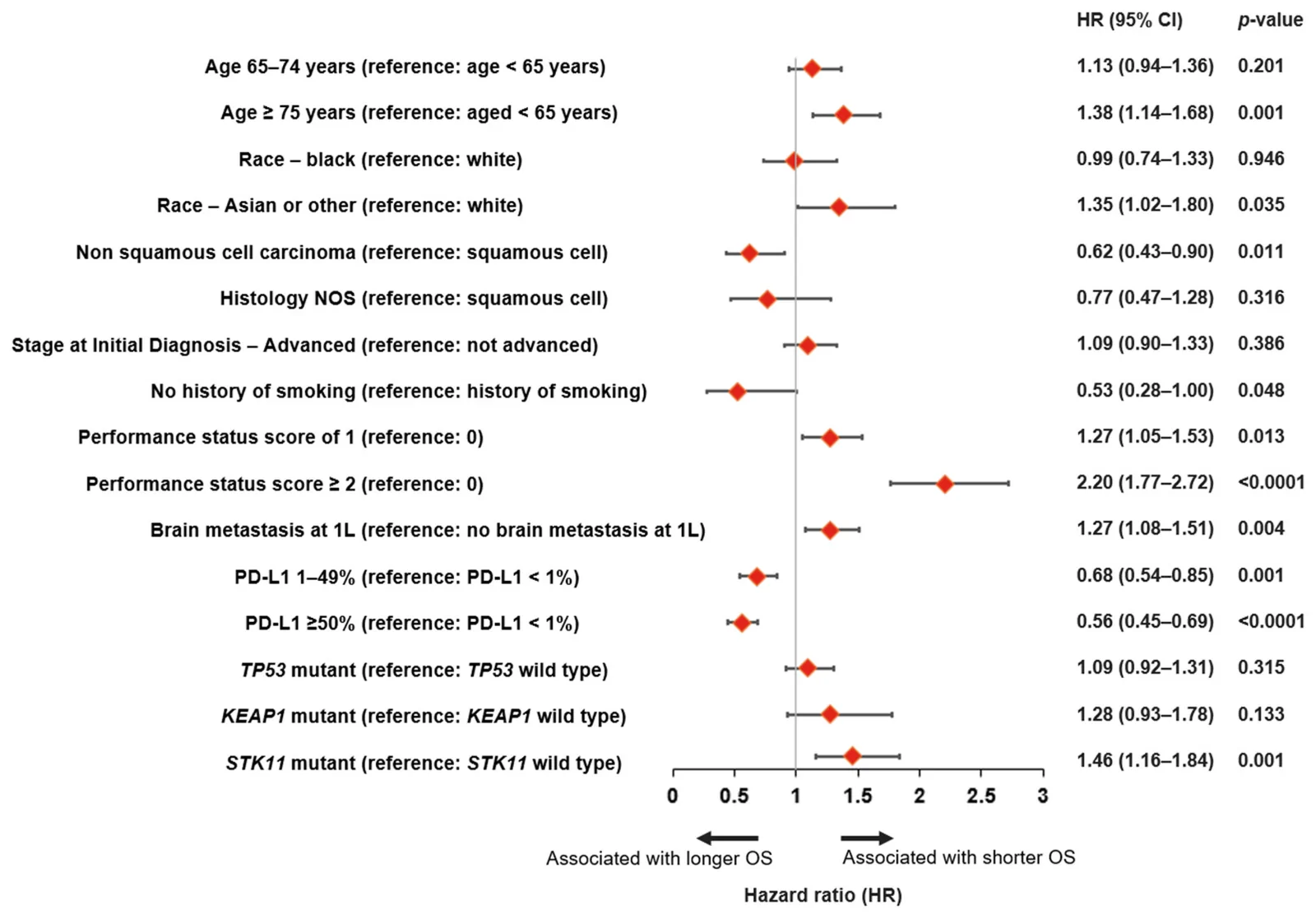
Forest plot for variables associated with overall survival in patients with *KRAS* G12C-mutant NSCLC based on multivariable Cox proportional hazards regression model. Abbreviations: 1L, first-line; CI, confidence interval; HR, hazard ratio; *KRAS*, Kirsten rat sarcoma viral oncogene homolog; NOS, not otherwise specified; NSCLC, non-small-cell lung cancer; OS, overall survival; PD-L1, Programmed Cell Death Ligand 1.

Adjusted associations between demographic and clinical characteristics and OS among patients with *KRAS* G12C-mutant NSCLC were evaluated with multivariable Cox modeling (EHR cohort; n = 1227—Figure 5). Patient characteristics significantly associated with shorter OS included age ≥ 75 years (HR 1.38, 95% CI 1.14, 1.68), Asian/Other or unknown race (HR 1.35, 95% CI 1.02, 1.80; HR 1.27, 95% 1.03, 1.56), performance status score of ≥2 (HR 2.20, 95% CI 1.77, 2.72), and brain metastases (HR 1.27, 95% CI 1.08, 1.51). STK11 mutations were significantly associated with shorter OS (HR 1.46, 95% CI 1.16, 1.84), but KEAP1 and TP53 mutations were not. Patients with tumors with PD-L1 1–49% (HR 0.68, 95% CI 0.54, 0.85) and PD-L1 ≥ 50% (HR 0.56, 95% CI 0.45, 0.69) had significantly longer OS compared with patients with tumors with PD-L1 <1%.

### 3.5. Outcomes for Patients Treated with Platinum-Based Chemotherapy Plus Pembrolizumab by KRAS Status (CGDB and EHR Databases)

**Figure 6:**
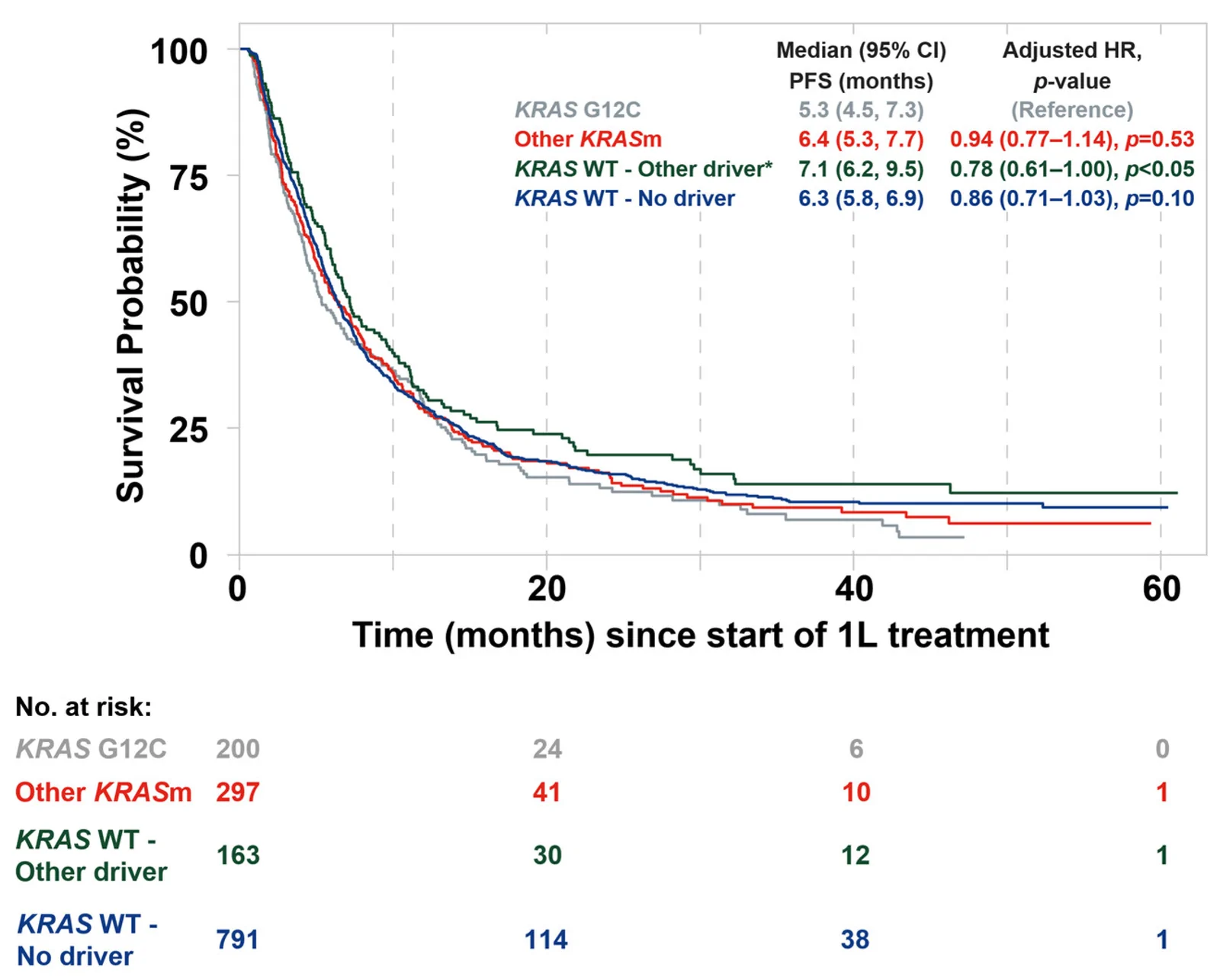
Progression-free survival by *KRAS* mutation status for patients treated with first-line platinum-based chemotherapy plus pembrolizumab—CGDB. Note: Adjusted HR and *p*-value from multivariable Cox proportional hazards regression model including age, sex, race, histology, performance status, stage at initial diagnosis, smoking history, PD-L1 expression, brain metastasis, STK11 mutation, KEAP1 mutation, and TP53 mutation. * “KRAS WT—Other driver” group included patients with EGFR, ALK, MET, BRAF, ROS1, NTRK, or RET alterations. Abbreviations: 1L, first-line; ALK, anaplastic lymphoma kinase; BRAF, B-Raf proto-oncogene, serine/threonine kinase; CGDB, Clinico-Genomic Database; CI, confidence interval; EGFR, epidermal growth factor receptor; HR, hazard ratio; MET, MET proto-oncogene, receptor tyrosine kinase; NTRK, neurotrophic tyrosine receptor kinase; PD-L1, Programmed Cell Death Ligand 1; PFS, progression-free survival; RET, rearranged during transfection; ROS1, ROS pro-to-oncogene 1, receptor tyrosine kinase; WT, wild type.

**Figure 7:**
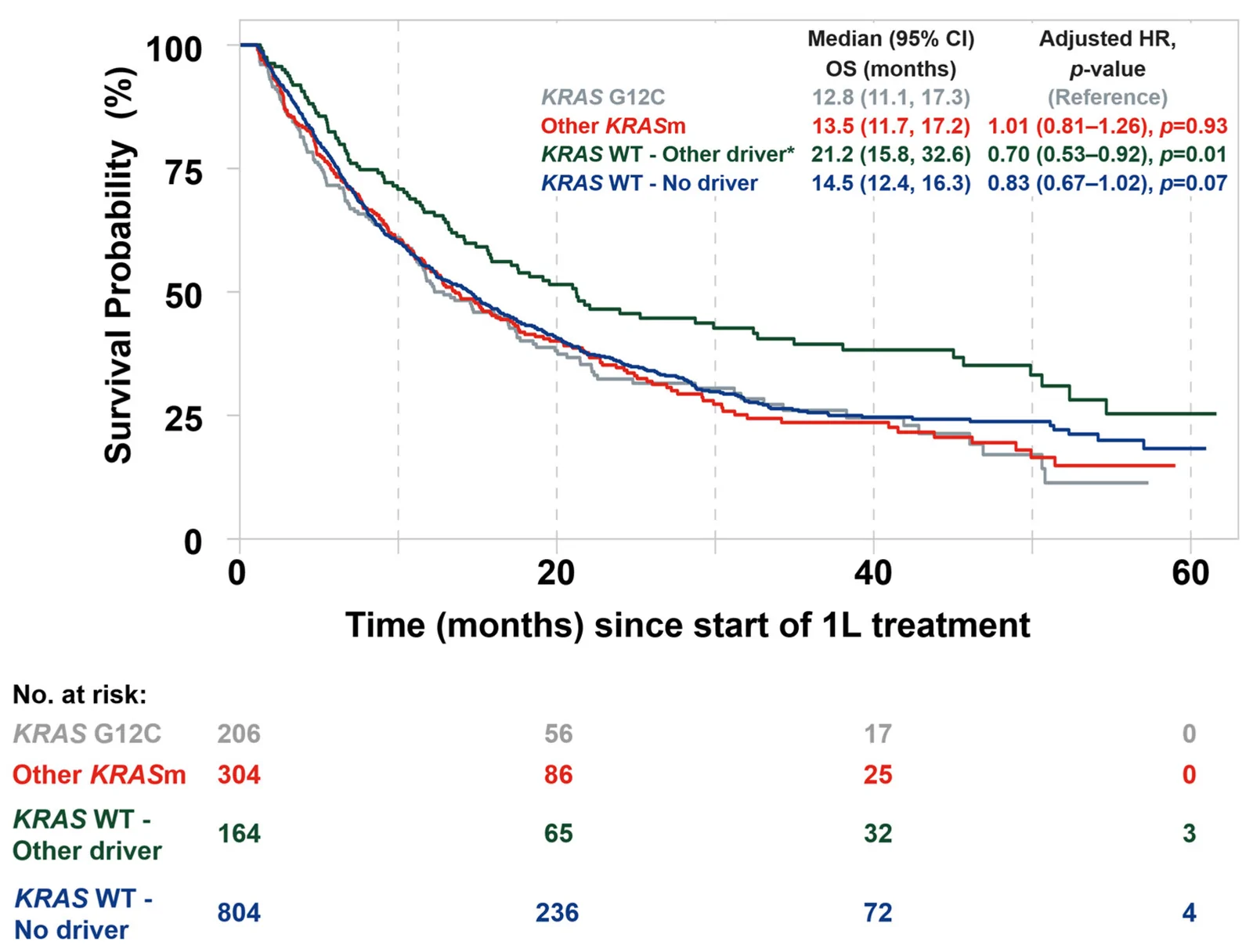
Overall survival by *KRAS* mutation status for patients treated with first-line platinum-based chemotherapy plus pembrolizumab—CGDB. Note: Adjusted HR and *p*-value from multivariable Cox proportional hazards regression model including age, sex, race, histology, performance status, stage at initial diagnosis, smoking history, PD-L1 expression, brain metastasis, STK11 mutation, KEAP1 mutation, and TP53 mutation. * “KRAS WT—Other driver” group included patients with EGFR, ALK, MET, BRAF, ROS1, NTRK, or RET alterations. Abbreviations: 1L, first-line; ALK, anaplastic lymphoma kinase; BRAF, B-Raf proto-oncogene, serine/threonine kinase; CGDB, Clinico-Genomic Database; CI, confidence interval; EGFR, epidermal growth factor receptor; HR, hazard ratio; MET, MET proto-oncogene, receptor tyrosine kinase; NTRK, neurotrophic tyrosine receptor kinase; PD-L1, Programmed Cell Death Ligand 1; RET, rearranged during transfection; ROS1, ROS proto-oncogene 1, receptor tyrosine kinase; WT, wild type.

In the CGDB, patients with *KRAS* G12C-mutant NSCLC treated with platinum-based chemotherapy plus pembrolizumab had median PFS of 5.28 months (95% CI: 4.52, 7.25) (Figure 6). Median PFS estimates were similar across subgroups of patients treated with platinum-based chemotherapy plus pembrolizumab: 6.43 months (95% CI: 5.28, 7.67) for other *KRAS* mutations, 6.33 months (95% CI: 5.84, 6.92) for *KRAS* wild type with no driver mutations, and 7.11 months (95% CI: 6.16, 9.54) for *KRAS* wild type with other driver mutations. Median OS was 12.82 months (95% CI: 11.11, 17.34) for patients with *KRAS* G12C mutations, 13.54 months (95% CI: 11.67, 17.18) for other *KRAS* mutations, 14.46 months (95% CI: 12.36, 16.26) for patients with *KRAS* wild type and no driver mutations, and 21.18 months (95% CI: 15.77, 32.62) for patients with *KRAS* wild type and other driver mutations (Figure 7). In multivariable models adjusting for demographic and clinical variables, PFS and OS were longer for the *KRAS* wild type with driver mutations group compared with the *KRAS* G12C group (HR = 0.78, 95% CI 0.61, 1.00; HR = 0.70, 95% CI 0.53, 0.92) (Figure 6 and Figure 7).

**Figure 8:**
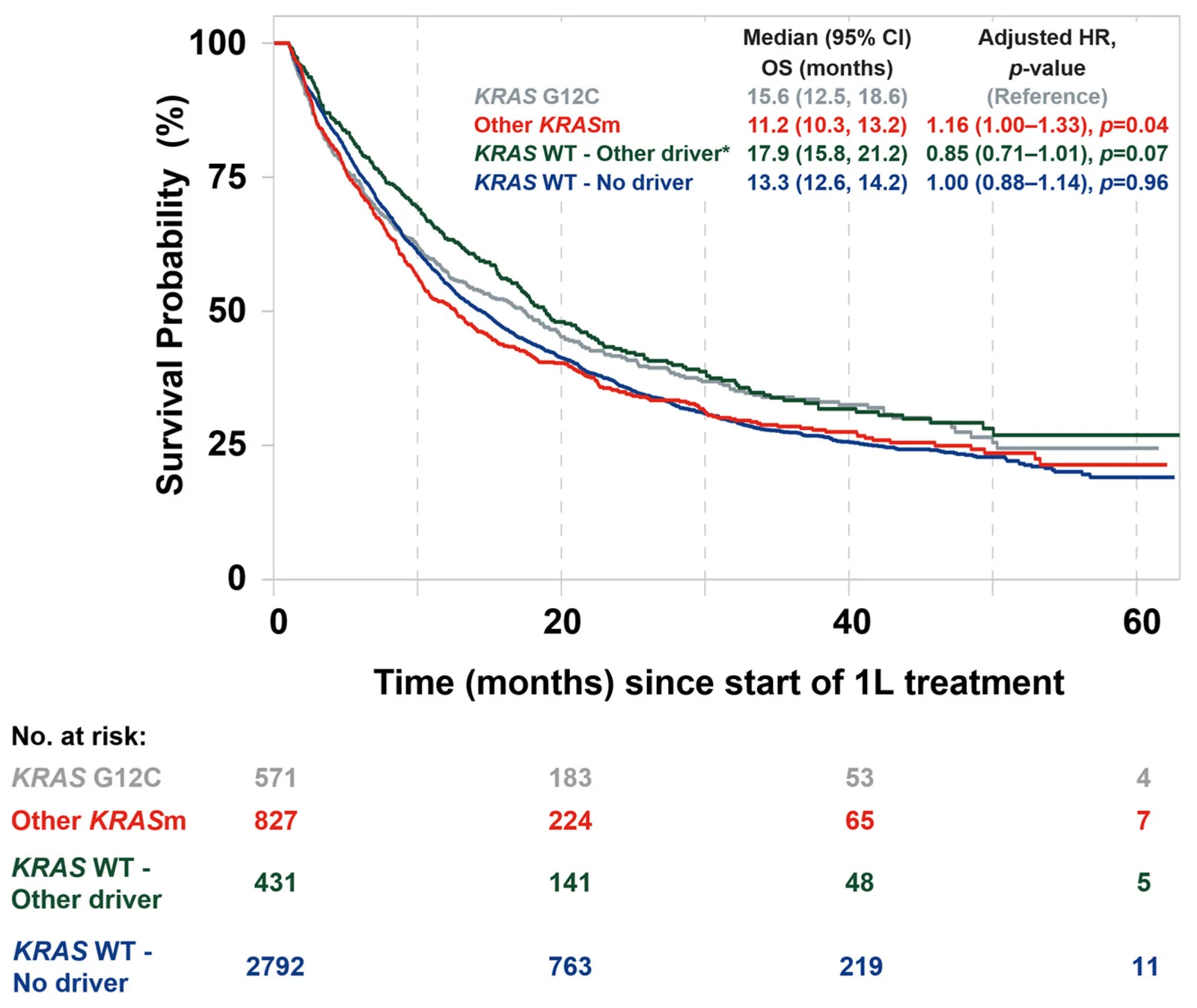
Overall survival by *KRAS* status for patients treated with first-line platinum-based chemotherapy plus pembrolizumab—EHR database. Note: Adjusted HR and *p*-value from multivariable weighted Cox regression model including age, sex, race, histology, performance status, stage at initial diagnosis, smoking history, PD-L1 expression, brain metastasis, STK11 mutation, KEAP1 mutation, and TP53 mutation. * “KRAS WT—Other driver” group included patients with EGFR, ALK, MET, BRAF, ROS1, NTRK, or RET alterations. Abbreviations: 1L, first-line; ALK, anaplastic lymphoma kinase; BRAF, B-Raf proto-oncogene, serine/threonine kinase; CI, confidence interval; EGFR, epidermal growth factor receptor; EHR, electronic health record; HR, hazard ratio; MET, MET proto-oncogene, receptor tyrosine kinase; NTRK, neurotrophic tyrosine receptor kinase; OS, overall survival; PD-L1, Programmed Cell Death Ligand 1; RET, rearranged during transfection; ROS1, ROS proto-oncogene 1, receptor tyrosine kinase; WT, wild type.

In the EHR database, median OS was 15.57 months (95% CI: 12.52, 18.62) for patients with *KRAS* G12C-mutant NSCLC, 11.21 months (95% CI: 10.30, 13.21) for other *KRAS*-mutant NSCLC, 13.31 months (95% CI: 12.56, 14.23) for *KRAS* wild type with no driver mutations, and 17.93 months (95% CI: 15.80, 21.18) for *KRAS* wild type with driver mutations (Figure 8). In the multivariable model, patients with other *KRAS* mutations had shorter OS compared with patients with *KRAS* G12C mutations (Figure 8).

### 3.6. Outcomes for Patients with PD-L1 ≥ 50% Treated with First-Line Pembrolizumab Monotherapy by KRAS Status (CGDB and EHR Data)

**Figure 9:**
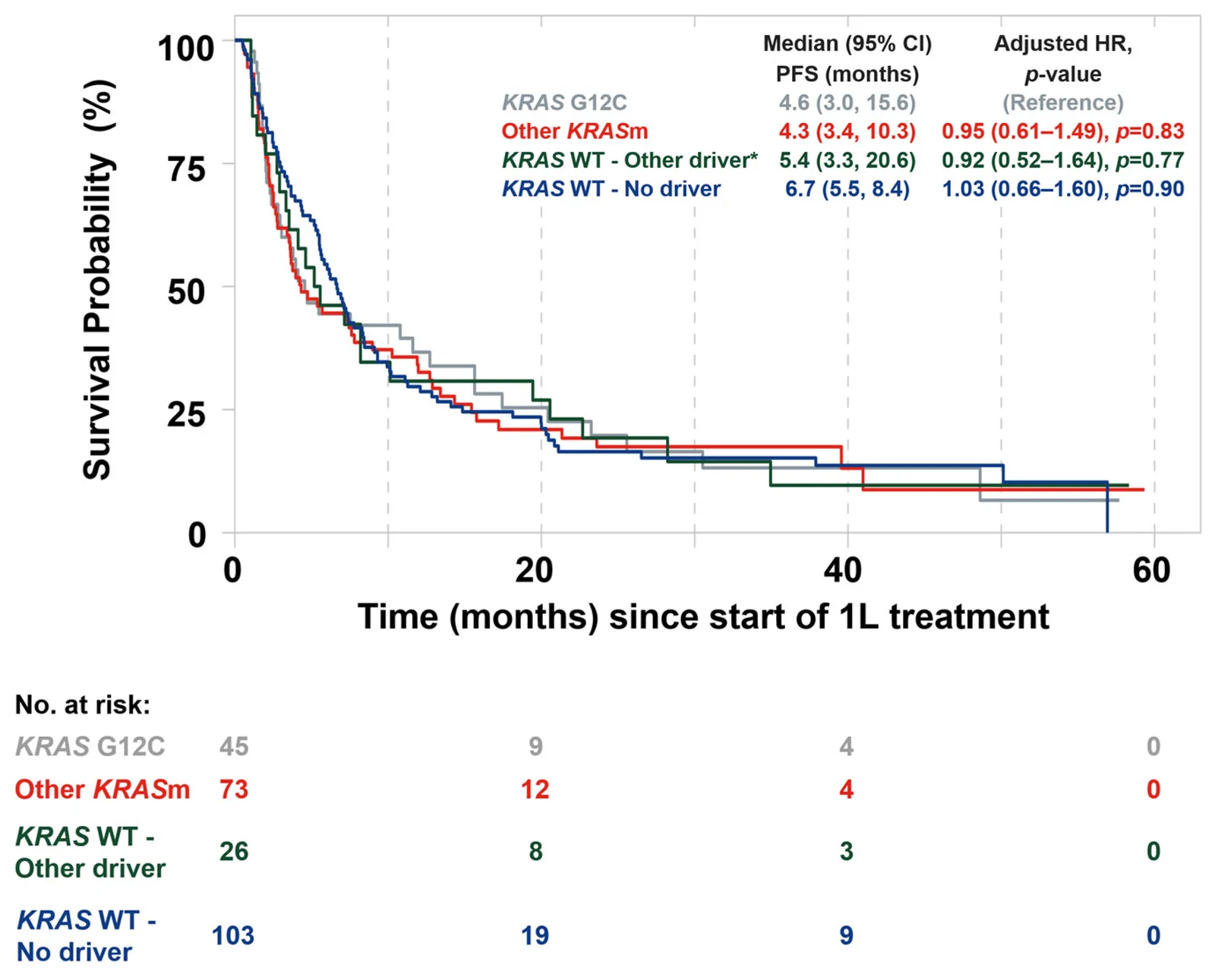
Progression-free survival by *KRAS* mutation status for patients with PD-L1 ≥ 50% treated with first-line pembrolizumab monotherapy—CGDB. Note: Adjusted HR and *p*-value from multivariable Cox proportional hazards regression model including age, sex, race, histology, performance status, stage at initial diagnosis, smoking history, brain metastasis, STK11 mutation, KEAP1 mutation, and TP53 mutation. * “KRAS WT—Other driver” group included patients with EGFR, ALK, MET, BRAF, ROS1, NTRK, or RET alterations. Abbreviations: 1L, first-line; ALK, anaplastic lymphoma kinase; BRAF, B-Raf proto-oncogene, serine/threonine kinase; CGDB, Clinico-Genomic Database; CI, confidence interval; EGFR, epidermal growth factor receptor; HR, hazard ratio; MET, MET proto-oncogene, receptor tyrosine kinase; NTRK, neurotrophic tyrosine receptor kinase; PD-L1, Programmed Cell Death Ligand 1; PFS, progression-free survival; RET, rearranged during transfection; ROS1, ROS proto-oncogene 1, receptor tyrosine kinase; WT, wild type.

**Figure 10:**
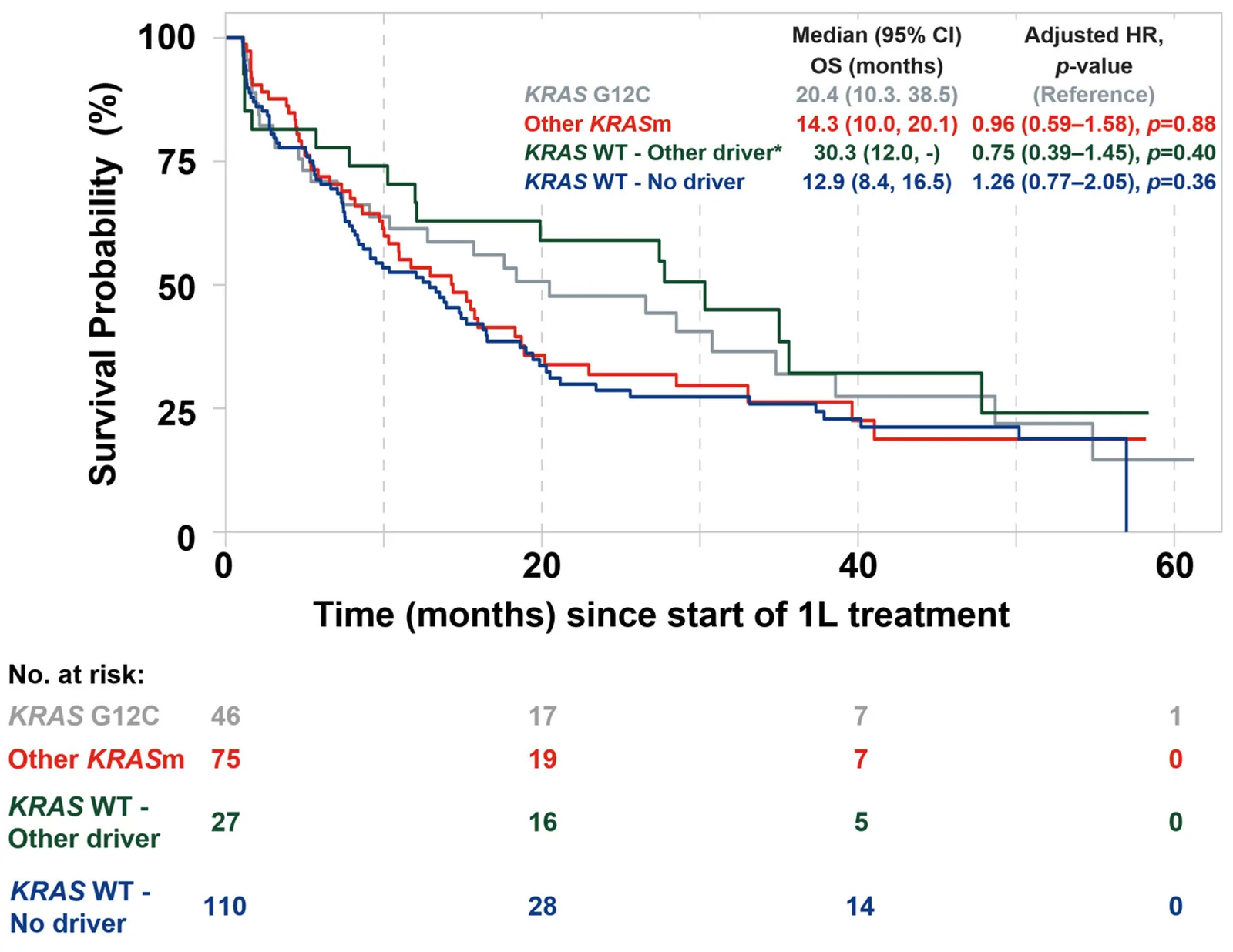
Overall survival by *KRAS* status for patients with PD-L1 ≥ 50% treated with first-line pembrolizumab monotherapy—CGDB. Note: Adjusted HR and *p*-value from multivariable weighted Cox regression model including age, sex, race, histology, performance status, stage at initial diagnosis, smoking history, brain metastasis, STK11 mutation, KEAP1 mutation, and TP53 mutation. * “KRAS WT—Other driver” group included patients with EGFR, ALK, MET, BRAF, ROS1, NTRK, or RET alterations. Abbreviations: 1L, first-line; ALK, anaplastic lymphoma kinase; BRAF, B-Raf proto-oncogene, serine/threonine kinase; CI, confidence interval; EGFR, epidermal growth factor receptor; EHR, electronic health record; HR, hazard ratio; MET, MET proto-oncogene, receptor tyrosine kinase; NTRK, neurotrophic tyrosine receptor kinase; OS, overall survival; PD-L1, Programmed Cell Death Ligand 1; RET, rearranged during transfection; ROS1, ROS proto-oncogene 1, receptor tyrosine kinase; WT, wild type.

In the CGDB, patients with *KRAS* G12C-mutant NSCLC and PD-L1 ≥ 50% treated with pembrolizumab monotherapy had median PFS of 4.56 months (95% CI: 2.95, 15.64). Median PFS was similar across the other subgroups of patients with PD-L1 ≥ 50% treated with pembrolizumab: 4.33 months (95% CI: 3.41, 10.26) for other *KRAS* mutations, 6.66 months (95% CI: 5.51, 8.43) for *KRAS* wild type with no driver mutations, and 5.38 months (95% CI: 3.34, 20.56) for *KRAS* wild type with driver mutations (Figure 9). In the multivariable model, there were no statistically significant differences between groups (Figure 9). Median OS was 20.43 months (95% CI: 10.33, 38.52) for patients with *KRAS* G12C-mutant NSCLC, 14.33 months (95% CI: 9.97, 20.13) for patients with other *KRAS* mutations, 12.85 months (95% CI: 8.36, 16.49) for *KRAS* wild type with no driver mutations, and 30.26 months (95% CI: 12.03, NE) for *KRAS* wild type with other driver mutations (Figure 10). No statistically significant differences in OS were observed in the multivariable model.

**Figure 11:**
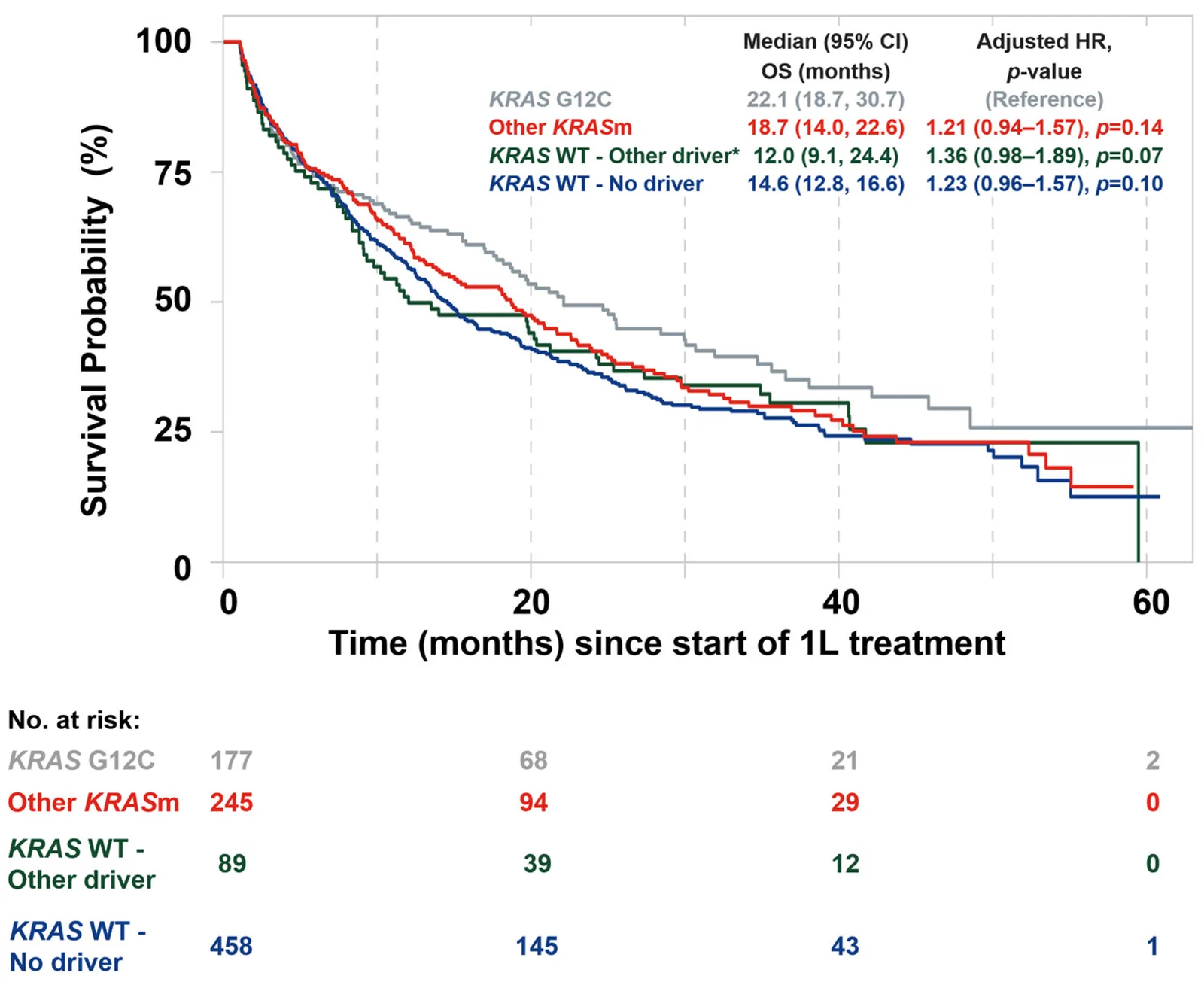
Overall survival by KRAS status for patients with PD-L1 ≥ 50% treated with first-line pembrolizumab monotherapy—EHR database. Note: Adjusted HR and *p*-value from multivariable Cox proportional hazards regression model including age, sex, race, histology, performance status, stage at initial diagnosis, smoking history, brain metastasis, STK11 mutation, KEAP1 mutation, and TP53 mutation. * “KRAS WT—Other driver” group included patients with EGFR, ALK, MET, BRAF, ROS1, NTRK, or RET alterations. Abbreviations: ALK, anaplastic lymphoma kinase; BRAF, B-Raf proto-oncogene, serine/threonine kinase; EGFR, epidermal growth factor receptor; EHR, electronic health record-driven; HR, hazard ratio; MET, MET proto-oncogene, receptor tyrosine kinase; NTRK, neurotrophic tyrosine receptor kinase; OS, overall survival; PD-L1, Programmed Cell Death Ligand 1; RET, rearranged during transfection; ROS1, ROS proto-oncogene 1, receptor tyrosine kinase; WT, wild type.

In the EHR database, median OS was 22.13 months (95% CI:18.66, 30.72) for patients with *KRAS* G12C-mutant NSCLC and PD-L1 ≥ 50% treated with pembrolizumab monotherapy. Results for the other subgroups are shown in Figure 11. There were no statistically significant differences between groups in the multivariable model. (See Tables S7–S10 for patient and tumor characteristics of the treatment groups in the EHR and CGDB).

## 4. Discussion

In this nationwide US study of patients with advanced *KRAS* G12C-mutant NSCLC, we evaluated clinical presentation, characteristics of patients treated with immunotherapy versus chemoimmunotherapy, predictors of OS, and outcomes for those treated with first-line immunotherapy and chemoimmunotherapy. Factors significantly associated with shorter OS in this patient population included PD-L1 < 1%, brain metastases at the start of first-line therapy, performance status score ≥ 2, and *STK11* co-mutation. Study results demonstrate an unmet need in patients with *KRAS* G12C-mutant NSCLC. Less than 40% of patients treated with first-line therapy received subsequent treatment. Overall survival ranged from 13 to 16 months for patients with *KRAS* G12C-mutant NSCLC treated with platinum-based chemotherapy plus pembrolizumab and from 20 to 22 months for patients with *KRAS* G12C-mutant NSCLC and PD-L1 ≥ 50% treated with pembrolizumab monotherapy.

Platinum-based chemotherapy plus pembrolizumab and pembrolizumab monotherapy are the currently preferred first-line treatments for patients with *KRAS* G12C-mutant NSCLC, with approximately two-thirds of patients in this study receiving these regimens. Results demonstrate that first-line treatment selection was guided by PD-L1 expression and clinical characteristics, in accordance with clinical practice guidelines [8,9,10]. Most patients (81.6%) treated with pembrolizumab monotherapy had PD-L1 expression ≥ 50%, and 43% had very high PD-L1 ≥ 90%. These patients were also older and more likely to have poor performance status compared with those receiving platinum-based chemotherapy plus pembrolizumab, indicating that pembrolizumab monotherapy may be preferred for patients who may be too frail to receive chemotherapy. Among patients treated with platinum-based chemotherapy plus pembrolizumab, 30.9% had PD-L1 expression <1%, 38.0% had PD-L1 expression of 1–49%, and 31.1% had PD-L1 ≥ 50%. These patients were more likely to have *STK11* and *KEAP1* co-mutations and bone metastases compared with patients treated with pembrolizumab monotherapy. Other prognostic features that may influence prescribing, such as comorbid conditions and symptomatic disease, were not captured in the EHR database. For patients with PD-L1 ≥ 50%, clinical practice guidelines include both immunotherapy and chemoimmunotherapy as preferred first-line regimens. However, an FDA pooled analysis of data from 12 registrational trials concluded that all patients appear to benefit from adding chemotherapy to immunotherapy, regardless of PD-L1 expression or *KRAS* status [38]. We expect such patients to have good performance status and to be able to tolerate chemotherapy in combination with immunotherapy. A recent study evaluating first-line therapy for advanced NSCLC reported that use of immunotherapy alone declined among patients with PD-L1 ≥ 50% from 2018 to 2024 in the United States [39,40].

Among patients with *KRAS* G12C-mutant NSCLC who received first-line treatment in this study, less than 40% received any subsequent treatment. Two KRAS G12C inhibitors, sotorasib and adagrasib, have received accelerated approval by FDA for patients with *KRAS* G12C-mutant locally advanced or metastatic NSCLC who have received at least one prior systemic therapy. However, our study results highlight that the majority of patients with *KRAS* G12C mutations are not able to benefit from these therapies because they never receive subsequent treatment. Among patients treated with second-line therapy in this study, approximately 30% received KRAS G12C inhibitors. The study period included several years prior to the approval of these drugs; therefore, more updated data post-approval will be needed to evaluate the uptake of this drug class among patients who receive second-line therapy and to compare the characteristics of patients who receive KRAS G12C inhibitors versus other second-line regimens.

In this study, 25% of patients had documented brain metastasis at the start of first-line therapy, and OS was significantly shorter in patients with brain metastasis. Previous studies of patients with *KRAS* G12C-mutant NSCLC have reported a prevalence of brain metastases ranging from 27 to 56%, with higher estimates for those reporting results among patients with confirmed assessment of intracranial disease by imaging (CT and/or MRI) [41,42,43,44,45]. A systematic review and meta-analysis reported a pooled baseline prevalence of 30.2% and yearly incidence of 10.0% for patients with *KRAS*-mutant NSCLC [46]. Information on brain imaging was not available in the database used for this study. Therefore, the frequency of documented brain metastasis likely underestimates the true prevalence because the analysis was not restricted to patients who underwent brain imaging. Other studies have also shown poor prognosis for patients with brain metastases. A study from the Dana Farber Cancer Institute demonstrated that OS in patients with *KRAS-*mutant NSCLC was significantly shorter among those with brain metastases at the time of metastatic diagnosis (13.2 months vs. 16.0 months, *p* = 0.017) and observed a similar trend, although not statistically significant, among patients with *KRAS* G12C-mutant NSCLC (16.1 months vs. 19.0 months, *p* = 0.063) [42]. The high prevalence of brain metastases and associated poor prognosis for these patients on the current standard of care treatments reinforces the need for targeted therapies with intracranial activity for patients with *KRAS* G12C-mutant NSCLC.

We evaluated PFS and OS for patients with *KRAS* G12C-mutant NSCLC treated with first-line platinum-based chemotherapy plus pembrolizumab and pembrolizumab alone. Results are generally consistent with several studies conducted in both the US and Europe that have reported median OS of 18–28 months for patients treated with immunotherapy [47,48], 16 months for patients treated with immunotherapy ± chemotherapy [49], and 9–16 months for patients treated with chemoimmunotherapy [47,50,51]. Patients without *KRAS* G12C mutations who received these regimens were included as comparison groups to provide context for the results. Outcomes were generally poor for all subgroups evaluated, and observed differences in PFS and OS between patients with *KRAS* G12C-mutant NSCLC and other subgroups may not be clinically meaningful. Previous studies have also demonstrated similar outcomes to immunotherapy-based treatments between patients with *KRAS* G12C-mutant NSCLC, other *KRAS* mutations, and *KRAS* wild-type disease [47,50,52]. However, broader analyses of patients with advanced NSCLC have shown that patients with oncogenic drivers with available approved targeted therapies had significantly longer OS than patients with *KRAS* G12C-mutant NSCLC (27 vs. 11 months, *p* < 0.001) [1].

*KRAS* G12C-mutant NSCLC is a heterogeneous disease. Co-mutations involving *STK11* and *KEAP1* were observed in the overall study population, and tumors with PD-L1 < 50% were enriched for these co-mutations, consistent with prior reports [47,53]. Co-mutations can alter the immune microenvironment, resulting in immunologically cold tumors [54]. *STK11* alterations are associated with a non-T cell-inflamed microenvironment exhibiting low infiltrating CD3+, CD4+, and CD8+ T cells and low tumor cell expression of PD-L1, and *KEAP1* alterations are associated with an immunosuppressive tumor microenvironment and low CD8+ T cell infiltration [54,55,56]. PD-L1 expression levels and *STK11* and *KEAP1* co-mutations may impact clinical benefit for patients with *KRAS* G12C-mutant NSCLC treated with standard of care immunotherapy-based treatment in the first-line setting. More intensive front-line approaches may be needed, such as the combination of KRAS G12C inhibitors with immune checkpoint inhibitors and/or chemotherapy to achieve deeper and more durable responses. *STK11* and *KEAP1* have been identified as biomarkers of immunotherapy resistance in metastatic NSCLC, particularly among patients with *KRAS*-mutant NSCLC [57]. *KEAP1* co-mutations also appear to confer resistance to chemotherapy and KRAS G12C inhibitors, in addition to immunotherapy. The current study and previous studies have identified *STK11* and *KEAP1* as independent negative prognostic factors among patients with *KRAS*-mutant NSCLC treated with immune-based therapy [50,58]. Negative associations between *STK11* and survival outcomes persist across PD-L1 strata or after controlling for PD-L1 status, suggesting that the relationship is independent of PD-L1 [47,53,57]. Evidence also suggests that co-occurring *STK11* and *KEAP1* mutations are associated with the worst prognosis [47,50,51,53,57] among patients with *KRAS*-mutant NSCLC treated with immunotherapy, as their co-occurrence imparts multiple mechanisms of therapeutic resistance [53]. Further adding to the complexity, *TP53* co-mutations are associated with an inflamed tumor immune microenvironment and increased PD-L1 expression in *KRAS*-mutant NSCLC [54] and may be associated with enhanced response to PD-L1 inhibitors and improved survival outcomes [13,54]. However, the impact of *TP53* mutations in KRAS-mutant NSCLC may depend on the presence of other co-mutations, with preliminary evidence showing worse outcomes in patients with TP53 mutations and *STK11* + *KEAP1* or *STK11* co-mutations but improved outcomes in those with *TP53* mutations and *KEAP1* co-mutations [59]. In the current study, we were unable to evaluate combinations of *STK11, KEAP1*, and *TP53* mutations, which is a limitation. Given testing practices during the study period, the cell sizes were small for multiple co-mutations, and a large proportion of patients were not tested for each mutation. Future studies will be needed to further explore this area. The potential prognostic and predictive roles of co-occurring mutations underscore the need for comprehensive testing, as knowledge of co-mutations may be integral to treatment decision-making for *KRAS* G12C-mutant NSCLC as more evidence emerges.

The strengths of this study included a large sample size of patients with *KRAS* G12C-mutant NSCLC treated with platinum-based chemotherapy plus pembrolizumab and pembrolizumab alone and recent data from more than 800 practices across the United States from 2018 to 2023. Results provide insights into first-line treatment selection for patients with *KRAS* G12C-mutant NSCLC by comparing the characteristics of patients treated with chemoimmunotherapy vs. immunotherapy and showing that most patients do not receive subsequent therapy, describing an important unmet need not highlighted in previous studies. Moreover, outcomes were analyzed separately for patients treated with platinum-based chemotherapy plus pembrolizumab and pembrolizumab alone (in PD-L1 ≥ 50%), in contrast to several previous studies that have described results for immunotherapy ± chemotherapy. Median PFS and OS estimates can provide a benchmark of real-world outcomes for the control arm regimens and PD-L1 groups currently being evaluated in first-line phase 3 trials of KRAS G12C inhibitors. The characterization of first-line treatment practices and outcomes in a large, real-world cohort of predominantly community practice patients is an important strength in a rapidly evolving therapeutic landscape to put into context data from multiple disclosures for KRAS G12C inhibitor assets that are available or expected in the 1L and/or 2L setting.

Demographic and clinical characteristics reported in this study, including age, sex, race, histology, and smoking status, are largely consistent with previous studies in the US. We observed a negative association between Asian/other race and OS, which has not been previously reported for patients with *KRAS* G12C-mutant NSCLC. Given small sample sizes, the Asian and other race categories were grouped together in this study, and the other race category was a heterogeneous group including American Indian or Alaskan Native, Native Hawaiian or other Pacific Islander, and race descriptions that fall in multiple race categories. Previous studies have shown that both self-reported race and genetic ancestry are associated with *KRAS* driver mutation and subtypes, with lower prevalence among patients with Asian [2,60] and Native Americans/admixed American ancestry [61]. Asian race was significantly associated with a lower likelihood of *KRAS*-mutant NSCLC compared to white patients, even after controlling for age, histology, sex, and smoking status [60]. A recent study in an advanced NSCLC population reported lower TMB in patients with Asian ancestry and showed that across all racial groups, higher TMB was associated with improved OS in patients treated with immunotherapy-based treatment [62]. The observed association between Asian/other race and OS in our study may reflect both genetic factors, as well as non-genetic factors such as comorbid conditions, access to healthcare resources and quality of care, treatment differences, or socioeconomic disparities. Further research is needed to evaluate the relationship between race/ethnicity and survival outcomes among patients with *KRAS* G12C-mutant NSCLC.

This study also had several limitations. Missing data in these databases reflect real-world biomarker testing and documentation practices. In addition, patients may not have documented sites of metastasis due to non-specific site descriptions in the documentation, lack of sufficient documentation to confirm a metastatic site, or a metastatic site outside of those included in the data model. Analyses evaluating documented brain metastases should be interpreted with caution, as the database did not capture whether patients had received assessment of intracranial disease by imaging. Patients with documented brain metastases in the EHR may have undergone imaging due to symptoms rather than for screening. Therefore, the prevalence of brain metastases was likely underestimated, and the comparison group for OS analyses may have contained patients with undetected and/or undocumented (asymptomatic) brain metastases. Multivariable models controlled for available demographic and clinical characteristics in the database; however, there is potential for residual unmeasured confounding. Small sample sizes and heterogeneity in the comparison groups for patients treated with pembrolizumab monotherapy resulted in variable outcomes and wide confidence intervals, limiting the power to detect statistically significant differences. Additionally, immunotherapy-based treatment is not recommended for patients with actionable driver mutations; therefore, results for this *KRAS* wild type patient subgroup should be interpreted with caution. Finally, given the rules used to assign line of therapy and regimen in this database, drugs that were added or switched to after 28 days are not reflected in the regimen name for the line of therapy.

## 5. Conclusions

*KRAS* G12C-mutant NSCLC is a heterogeneous disease, with PD-L1 expression and co-mutations influencing treatment decisions and clinical outcomes. Patients treated with platinum-based chemotherapy plus pembrolizumab and those with PD-L1 ≥50% and treated with pembrolizumab monotherapy had median overall survival of 13–16 months and 20–22 months, respectively. Less than 40% of patients advanced to second-line therapy. These results demonstrate “real-world” outcomes for patients with *KRAS* G12C-mutant NSCLC in clinical practice, providing a benchmark for outcomes on the current standard of care for these patients, and highlighting the significant unmet need for more efficacious targeted treatment options in the first-line setting. Chemoimmunotherapy and immunotherapy are the backbone of first-line treatment options for patients today, but earlier intervention with KRAS G12C inhibitors combined with current standard of care regimens or in novel combination strategies may improve outcomes for these patients.

## Figures and Tables

**Table 1:** Demographics and clinical characteristics of patients with *KRAS* G12C-mutant NSCLC.

Variable	EHR N = 1227	CGDB N = 447
Age—median (Q1–Q3), years	69 (63–76)	70 (63–77)
<55 years, n (%)	70 (5.7)	28 (6.3)
55–64 years, n (%)	309 (25.2)	110 (24.6)
65–74 years, n (%)	476 (38.8)	156 (34.9)
≥75 years, n (%)	372 (30.3)	153 (34.2)
Female, n (%)	710 (57.9)	257 (57.5)
Race, n (%)		
White	878 (71.6)	307 (68.7)
Black	82 (6.7)	29 (6.5)
Asian/Other	96 (7.8)	61 (14.1)
Unknown	171 (13.9)	47 (10.5)
Histology, n (%)		
Non-squamous cell carcinoma	1133 (92.3)	411 (91.9)
NSCLC histology not specified	50 (4.1)	13 (2.9)
Squamous cell carcinoma	44 (3.6)	23 (5.1)
History of smoking: Yes, n (%)	1206 (98.3)	441 (98.7)
CNS or brain metastasis, n (%)	307 (25.0)	112 (25.1)
Stage at initial diagnosis, n (%)		
Advanced (Stage IIIB-IV)	990 (80.7)	340 (76.1)
Early-stage (Stage I-IIIA)	220 (17.9)	96 (21.5)
Not reported	17 (1.4)	11 (2.5)
Performance status, n (%)		
0	348 (28.4)	127 (28.4)
1	533 (43.4)	195 (43.6)
2+	228 (18.6)	87 (19.5)
Unknown	118 (9.6)	38 (8.5)
PD-L1 status, n (%)		
<1%	216 (23.7)	61 (22.1)
1–49%	289 (31.7)	90 (32.6)
≥50%	408 (44.7)	125 (45.3)
No evidence of testing	314	171
*TP53* mutation, n (%) ^1^	442 (50.8)	239 (53.5)
*KEAP1* mutation, n (%) ^1^	64 (12.1)	87 (19.5)
*STK11* mutation, n (%) ^1^	168 (22.2)	122 (27.3)

^1^ Percentages based on patients with available test results. Abbreviations: 1L, first-line treatment; CGDB, Clinico-Genomic Database; CNS, central nervous system; EHR, electronic health record-driven; N, total number of patients in subgroup; n, number of patients in specific category; NSCLC, non-small-cell lung cancer; Q, quartile.

**Table 2:** First-line and second-line treatment regimens for patients with KRAS G12C-mutant NSCLC.

Treatment Regimens	EHR N = 1227	CGDB N = 447
Top 1L regimens, n (%)		
Overall		
Platinum-based chemotherapy + pembrolizumab	571 (46.5)	206 (46.1)
Pembrolizumab monotherapy	250 (20.4)	88 (19.7)
Carboplatin + paclitaxel	115 (9.4)	51 (11.4)
Carboplatin + pemetrexed	88 (7.2)	24 (5.4)
PD-L1 ≥ 50%	EHR n = 408	CGDB n = 125
Pembrolizumab monotherapy	177 (43.4)	46 (36.8)
Platinum-based chemotherapy + pembrolizumab	126 (30.9)	38 (30.4)
Carboplatin + paclitaxel	29 (7.1)	18 (14.4)
Carboplatin + pemetrexed	24 (5.9)	5 (4.0)
Top 2L regimens, n (%)	EHR n = 483	CGDB n = 167
Overall		
*KRAS* G12C inhibitor	144 (29.8)	48 (28.7)
Platinum-based chemotherapy + pembrolizumab	72 (14.9)	19 (11.4)
Pembrolizumab monotherapy	52 (10.8)	16 (9.6)
Docetaxel + ramucirumab	29 (6.0)	11 (6.6)

Abbreviations: 1L, first-line treatment; 2L, second-line treatment; CGDB, Clinico-Genomic Database; EHR, electronic health record-driven; N, total number of patients in subgroup; n, number of patients in specific category; NSCLC, non-small-cell lung cancer.

**Table 3:** Demographics and clinical characteristics of patients with *KRAS* G12C-mutant NSCLC treated with platinum-based chemotherapy plus pembrolizumab versus pembrolizumab monotherapy (EHR database).

	Platinum-Based Chemotherapy + Pembrolizumab N = 571	Pembrolizumab Monotherapy N = 250	*p*-Value
Age in years at 1L, n (%)			<0.0001
<55	41 (7.2)	8 (3.2)	
55–64	160 (28.0)	41 (16.4)	
65–74	231 (40.5)	84 (33.6)	
≥75	139 (24.3)	117 (46.8)	
Gender, n (%)			
Female	317 (55.5)	158 (63.2)	0.0402
Race, n (%)			
White	399 (69.9)	185 (74.0)	0.4124
Black	44 (7.7)	13 (5.2)	
Asian/Other	46 (8.1)	19 (8.0)	
Unknown race	82 (14.4)	32 (12.8)	
Histology, n (%)			0.038
Non-squamous	543 (95.1)	226 (90.4)	
Squamous	13 (2.3)	12 (4.8)	
Histology not specified	15 (2.6)	12 (4.8)	
Stage at initial diagnosis, n (%)			0.0195
Advanced stage (Stage IIIB-IV)	491 (86.0)	196 (78.4)	
Early stage (Stage I-IIIA)	73 (12.8)	51 (20.4)	
Stage not reported	7 (1.2)	3 (1.2)	
Smoking status, n (%)			0.527
History of smoking	563 (98.6)	245 (98.0)	
No history of smoking	8 (1.4)	5 (2.0)	
Smoking not documented	0 (0.0)	0 (0.0)	
ECOG Performance status, n (%)			0.0037
0	161 (28.2)	57 (22.8)	
1	257 (45.0)	104 (41.6)	
2+	99 (17.3)	69 (27.6)	
Unknown	54 (9.5)	20 (8.0)	
PD-L1 status, n (%) ^1^			<0.0001
<1%	125 (30.9)	10 (4.6)	
1–49%	154 (38.0)	30 (13.8)	
≥50%	126 (31.1)	177 (81.6)	
Unknown/untested	166	33	
Documented Sites of Metastases at 1L Treatment, n (%)			
Any sites of metastases	456 (79.9)	182 (72.8)	0.0253
Adrenal metastasis	101 (17.7)	37 (14.8)	0.3085
Bone metastasis	274 (48.0)	87 (34.8)	0.0005
Brain metastasis	161 (28.2)	77 (30.8)	0.4492
Liver metastasis	95 (16.6)	31 (12.4)	0.1211
Pleural metastasis	128 (22.4)	43 (17.2)	0.0903
*STK11* mutations ^1^, n (%)			0.0006
Mutant	88 (23.8)	16 (10.6)	
Wild type	282 (76.2)	135 (89.4)	
Unknown/untested	201	99	
*KEAP1* mutations ^1^, n (%)			0.1608
Mutant	34 (12.9)	7 (7.5)	
Wild type	229 (87.1)	86 (92.5)	
Unknown/untested	308	157	
*TP53* mutation ^1^, n (%)			0.9344
Mutant	212 (49.6)	95 (54.0)	
Wild type	215 (50.4)	81 (46.0)	
Unknown/untested	144	74	
Practice type, n (%)			0.0458
Academic	82 (14.4)	53 (21.2)	
Community	473 (82.8)	192 (76.8)	
Both	16 (2.8)	5 (2.0)	

^1^ Percentages based on patients with available test results. Abbreviations: 1L, first-line treatment; ECOG, Eastern Cooperative Oncology Group; EHR, electronic health record-driven; N, total number of patients in subgroup; n, number of patients in specific category; NSCLC, non-small-cell lung cancer; Q, quartile.

## Data Availability

The study utilized two US-based datasets: Flatiron Health-Foundation Medicine NSCLC Clinico-Genomic Database (FH-FMI CGDB) and the electronic health record-derived deidentified Flatiron Health Research Database (EHR database). Restrictions may apply to the availability of these data, which were used under agreement for this study and are, therefore, not publicly available. Requests for more information on data availability and licensing may be sent to Flatiron Health.

## Supplementary Materials

The following supporting information can be downloaded at https://www.mdpi.com/article/10.3390/cancers18172869/s1: Figure S1: Overall survival in patients with KRAS G12C-mutant NSCLC treated with platinum-based pembrolizumab plus chemotherapy by PD-L1 expression (EHR database); Table S1: KRAS G12C cohort selection; Table S2: Counts of patients treated with 1L platinum-based chemotherapy plus pembrolizumab and pembrolizumab monotherapy; Table S3: PD-L1 staining percent for patients with KRAS G12C-mutant NSCLC treated with platinum-based chemotherapy plus pembrolizumab versus pembrolizumab monotherapy (EHR database); Table S4: Demographic and clinical characteristics of patients with KRAS G12C-mutant NSCLC by PD-L1 expression—EHR database; Table S5: Demographic and clinical characteristics of patients with KRAS G12C-mutant NSCLC by PD-L1 expression—CGDB; Table S6: Demographic and clinical characteristics of patients with KRAS G12C-mutant NSCLC and PD-L1 ≥ 50% treated with platinum-based chemotherapy plus pembrolizumab versus pembrolizumab monotherapy (EHR database); Table S7: Demographic and clinical characteristics by KRAS mutation type of patients with NSCLC treated with 1L platinum-based chemotherapy plus pembrolizumab (EHR Database); Table S8: Demographic and clinical characteristics by KRAS mutation type of patients with NSCLC PD-L1 ≥ 50% treated with 1L pembrolizumab monotherapy (EHR database); Table S9: Patient characteristics by KRAS mutation of patients treated with 1L platinum-based chemotherapy plus pembrolizumab (CGDB); Table S10: Patient characteristics by KRAS mutation of patients with PD-L1 ≥ 50% treated with 1L pembrolizumab monotherapy (CGDB).

## Abbreviations

The following abbreviations are used in this manuscript:

Abbreviation	Definition
1L	first-line
2L	second-line
ALK	anaplastic lymphoma kinase
ASCO	American Society of Clinical Oncology
BRAF	B-Raf proto-oncogene, serine/threonine kinase
CC BY	Creative Commons Attribution
CGDB	Clinico-Genomic Database
CI	confidence interval
CNS	central nervous system
CT	computed tomography
ECOG	Eastern Cooperative Oncology Group
EGFR	epidermal growth factor receptor
EHR	electronic health record
ESMO	European Society for Medical Oncology
FDA	Food and Drug Administration
FH-FMI CGDB	Flatiron Health-Foundation Medicine non-small-cell lung cancer Clinico-Genomic Database
FMI	Foundation Medicine, Inc.
HR	hazard ratio
IASLC	International Association for the Study of Lung Cancer
ICI	immune checkpoint inhibitor
KEAP1	Kelch-like ECH-associated protein 1
KRAS	Kirsten rat sarcoma viral oncogene homolog
MET	MET proto-oncogene, receptor tyrosine kinase
MRI	magnetic resonance imaging
N	total number of patients in subgroup
n	number of patients in specific category
NA	not applicable
NCCN	National Comprehensive Cancer Network
NE	not estimable
NOS	not otherwise specified
NSCLC	non-small-cell lung cancer
NTRK	neurotrophic tyrosine receptor kinase
OS	overall survival
PD-1	programmed cell death protein 1
PD-L1	programmed cell death ligand 1
PFS	progression-free survival
PS	performance status
Q	quartile
Q1	first quartile
Q3	third quartile
RET	rearranged during transfection
ROS1	ROS proto-oncogene 1, receptor tyrosine kinase
rwPFS	real-world progression-free survival
SAS	Statistical Analysis System
STK11	serine/threonine kinase 11
TMB	tumor mutational burden
TPS	tumor proportion score
TP53	tumor protein p53
US	United States
WCLC	World Conference on Lung Cancer
WT	wild type
