# Supplementary material for: Real-World Treatment Patterns and Clinical Outcomes After First-Line Therapy in Patients with KRAS G12C-Mutant Advanced Non-Small-Cell Lung Cancer in the United States
Source: Cancers (Basel). 2026 Sep 4;18(17):2869. doi: 10.3390/cancers18172869 (PMC13565047; doi:10.3390/cancers18172869)
Supplement: Supplementary file 1 [file cancers-18-02869-s001.zip › cancers-4414073-supplementary.pdf]

Real-World Treatment Patterns and Clinical Outcomes after First-Line Therapy in Patients with *KRAS* G12C-Mutant Advanced Non-Small Cell Lung Cancer in the United States

Table of Contents

Supplementary Figure

1. Figure S1: Overall Survival in patients with *KRAS* G12C-mutant NSCLC treated with platinum-based pembrolizumab plus chemotherapy by PD-L1 expression (EHR database)

Supplementary Tables

1. Table S1: *KRAS* G12C Cohort Selection
2. Table S2: Counts for Patients Treated with 1L Platinum-based Chemotherapy plus Pembrolizumab and Pembrolizumab Monotherapy
3. Table S3: PD-L1 staining percent for patients with *KRAS* G12C-mutant NSCLC treated with platinum-based chemotherapy plus pembrolizumab versus pembrolizumab monotherapy (EHR database)
4. Table S4: Demographic and clinical characteristics for patients with *KRAS* G12C-mutant NSCLC by PD-L1 expression – EHR database
5. Table S5: Demographic and clinical characteristics for patients with *KRAS* G12C-mutant NSCLC by PD-L1 expression – CGDB
6. Table S6: Demographic and clinical characteristics in patients with *KRAS* G12C-mutant NSCLC and PD-L1 ≥ 50% treated with platinum-based chemotherapy plus pembrolizumab versus pembrolizumab monotherapy (EHR database)
7. Table S7: Demographic and clinical characteristics by *KRAS* mutation type in patients with NSCLC treated with 1L platinum-based chemotherapy plus pembrolizumab (EHR Database)
8. Table S8: Demographic and clinical characteristics by *KRAS* mutation type in patients with NSCLC PD-L1 ≥ 50% treated with 1L pembrolizumab monotherapy (EHR database)
9. Table S9: Patient characteristics by *KRAS* mutation in patients treated with 1L platinum-based chemotherapy plus pembrolizumab (CGDB)
10. Table S10: Patient characteristics by *KRAS* Mutation in patients with PD-L1 ≥ 50% treated with 1L pembrolizumab monotherapy (CGDB)

Supplementary Figure

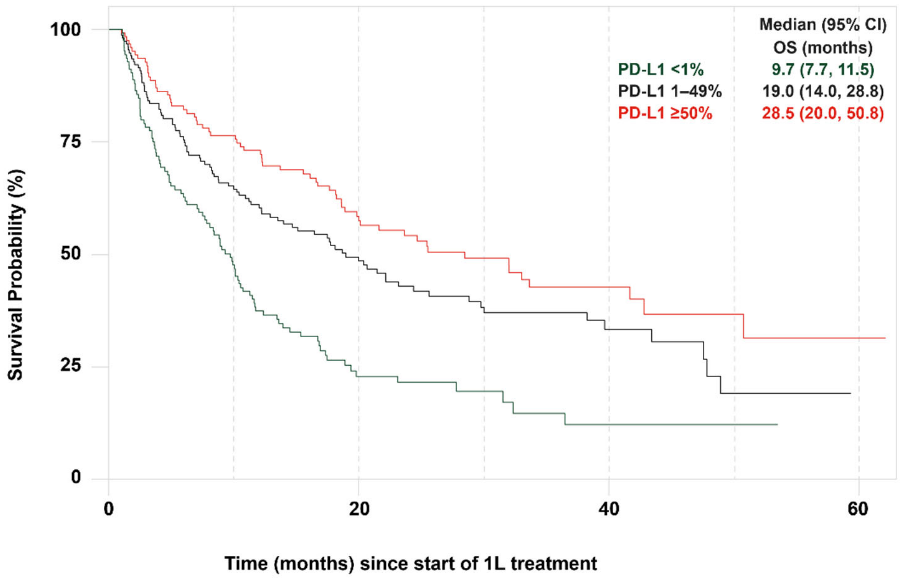

No. at risk:

PD-L1 <1%	125	19	3	0
PD-L1 1–49%	154	57	18	1
PD-L1 50%	126	58	17	2

Figure S1: Overall Survival in patients with *KRAS* G12C-mutant NSCLC treated with platinum-based pembrolizumab plus chemotherapy by PD-L1 expression (EHR database)

Abbreviations: KRAS, Kirsten rat sarcoma viral oncogene homolog; NSCLC, non-small cell lung cancer; OS, overall survival; PD-L1, Programmed Cell Death Ligand 1.

Supplementary Tables

Table S1: KRAS G12C Cohort Selection

**Flatiron Health Research Database – EHR Database**	**N**	**Flatiron Health Foundation Medicine Clinicogenomic Database - CGDB**	**N**
Diagnosed with NSCLC, at least 2 documented clinic visits occurring on or after 1/1/2011 (criteria for inclusion in overall database)	94,037	Diagnosed with NSCLC, at least 2 documented clinic visits occurring on or after 1/1/2011, and NGS testing by FMI (criteria for inclusion in overall database)	21,980
Stage IIIB, IIIC, IV or recurrent metastatic disease (required for inclusion in overall database)	94,037	Stage IIIB, IIIC, IV or recurrent metastatic disease	19,048
Started 1L therapy between 8/1/2018 and 12/31/2022	25,989	Started 1L therapy between 8/1/2018 and 12/31/2022	8,329
KRAS mutation test results ≤ 90 days before to ≤ 30 days after 1L start	13,927	NGS test with report date ≤ 90 days before to ≤ 30 days after 1L start	4,228
Exclude patients receiving Clinical Study Drug in 1L or 2L	13,072	Exclude patients receiving Clinical Study Drug in 1L or 2L	3,999
Exclude patients with date of death ≤ 30 days after 1L start	12,278	Exclude patients with date of death ≤ 30 days after 1L start	3,768
Patients with KRAS G12C mutant NSCLC	1,227	Patients with KRAS G12C mutant NSCLC	447

Abbreviations: CGDB, Clinico-Genomic Database; EHR, electronic health record-driven; FMI, Foundation Medicine, Inc.; *KRAS*, Kirsten rat sarcoma viral oncogene homolog; N, total number of patients in subgroup; NGS, next-generation sequencing; NSCLC, non-small cell lung cancer; 1L, first-line treatment; 2L, second-line treatment.

Table S2: Counts for Patients Treated with 1L Platinum-based Chemotherapy plus Pembrolizumab and Pembrolizumab Monotherapy

EHR Database	Patients, N	CGDB	Patients, N
**Received 1L platinum-based chemotherapy and pembrolizumab**		**Received 1L platinum-based chemotherapy and pembrolizumab**	
KRAS G12C	571	KRAS G12C	206
Other KRAS	827	Other KRAS	304
KRAS WT w/ Driver Mutations	431	KRAS WT w/ Driver Mutations	164
KRAS WT w/ no Driver Mutations	2,792	KRAS WT w/ no Driver Mutations	804
**Received 1L pembrolizumab monotherapy and has PD-L1 ≥ 50%**		**Received 1L pembrolizumab monotherapy and has PD-L1 ≥ 50%**	
KRAS G12C	177	KRAS G12C	46
Other KRAS	245	Other KRAS	75
KRAS WT w/ Driver Mutations	89	KRAS WT w/ Driver Mutations	27
KRAS WT w/ no Driver Mutations	458	KRAS WT w/ no Driver Mutations	110

Abbreviations: CGDB, Clinico-Genomic Database; EHR, electronic health record-driven; *KRAS*, Kirsten rat sarcoma viral oncogene homolog; PD-L1, Programmed Cell Death Ligand 1; WT, wild type; 1L, first-line treatment.

Table S3: PD-L1 staining percent for patients with KRAS G12C-mutant NSCLC treated with platinum-based chemotherapy plus pembrolizumab versus pembrolizumab monotherapy (EHR database)

	**Platinum-based chemotherapy +pembrolizumab N=571**	**Pembrolizumab monotherapy N=250**	**P-value**
**PD-L1 percent staining** [1], n (%)			
0%	80 (19.8)	4 (1.8)	<.0001
<1%	45 (11.1)	6 (2.8)	
1%	28 (6.9)	7 (3.2)	
2-4%	11 (2.7)	2 (0.9)	
5-9%	25 (6.2)	4 (1.8)	
10-19%	28 (6.9)	2 (0.9)	
20-29%	28 (6.9)	8 (3.7)	
30-39%	21 (5.2)	5 (2.3)	
40-49%	13 (3.2)	2 (0.9)	
50-59%	17 (4.2)	16 (7.4)	
60-69%	11 (2.7)	21 (9.7)	
70-79%	12 (3.0)	26 (12.0)	
80-89%	23 (5.7)	21 (9.7)	
90-99%	43 (10.6)	62 (28.6)	
100%	20 (4.9)	31 (14.3)	
Unknown/untested	166	33	

Abbreviations: EHR, electronic health record-driven; *KRAS*, Kirsten rat sarcoma viral oncogene homolog; N, total number of patients in subgroup; n, number of patients in specific category; NSCLC, non-small cell lung cancer; PD-L1, Programmed Cell Death Ligand 1.

Table S4: Demographic and clinical characteristics for patients with KRAS G12C-mutant NSCLC by PD-L1 expression – EHR database

	PD-L1 < 50% N=505	PD-L1 ≥ 50% N=408	P-value
Age at 1L , n (%)			
< 55 years	34 (6.7)	17 (4.2)	0.25
55-64 years	131 (25.9)	100 (24.5)	
65-74 years	196 (38.8)	158 (38.7)	
≥ 75 years	144 (28.5)	133 (32.6)	
Gender, n (%)			0.57
Female	284 (56.2)	237 (58.1)	
Race , n (%)			0.44
White	359 (71.1)	297 (72.8)	
Black	36 (7.1)	23 (5.6)	
Asian/Other	39 (7.7)	27 (6.6)	
Unknown race	71 (14.1)	61 (15.0)	
Histology, n (%)			0.009
Non-squamous	480 (95.0)	369 (90.4)	
Squamous	15 (3.0)	16 (3.9)	
Histology not specified	10 (2.0)	23 (5.6)	
Stage at diagnosis , n (%)			0.06
Advanced stage (Stage IIIB-IV)	393 (77.8)	343 (84.1)	
Early stage (Stage I-IIIA)	105 (20.8)	61 (15.0)	
Stage not reported	7 (1.4)	4 (1.0)	
Smoking status , n (%)			0.1144
History of smoking	495 (98.0)	405 (99.3)	
No history of smoking	10 (2.0)	3 (0.7)	
Smoking not documented	0 (0.0)	0 (0.0)	
ECOG Performance status, n (%)			0.42
0	150 (29.7)	108 (26.5)	
1	216 (42.8)	178 (43.6)	
≥2	88 (17.4)	82 (20.1)	
Unknown	51 (10.1)	40 (9.8)	
Documented Sites of Metastases, n (%)			
Any sites of metastases at 1L	344 (68.1)	277 (67.9)	0.942
Adrenal metastasis at 1L	60 (11.9)	56 (13.7)	0.406
Bone metastasis at 1L	198 (39.2)	138 (33.8)	0.094
Brain metastasis at 1L	127 (25.1)	109 (26.7)	0.591
Liver metastasis at 1L	62 (12.3)	50 (12.3)	0.992

Pleural metastasis at 1L	82 (16.2)	74 (18.1)	0.448
Brain metastasis at any time	161 (31.9)	133 (32.6)	0.818
STK11 mutation among tested, n (%)			<0.0001
Mutant	109 (34.1)	18 (7.4)	
Wild type	211 (65.9)	226 (92.6)	
Unknown/untested	185	164	
KEAP1 mutation among tested, n (%)			0.0133
Mutant	38 (16.2)	14 (8.0)	
Wild type	196 (83.8)	161 (92.0)	
Unknown/untested	271	233	
TP53 mutation among tested, n (%)			0.0004
Mutant	166 (45.6)	167 (59.6)	
Wild type	198 (54.4)	113 (40.4)	
Unknown/untested	141	128	
Practice type, n (%)			0.854
Academic	66 (13.1)	55 (13.5)	
Community	425 (84.2)	344 (84.3)	
Both	14 (2.8)	9 (2.2)	

Abbreviations: 1L, first-line treatment; EHR, electronic health record-driven; KRAS, Kirsten rat sarcoma viral oncogene homolog; N, total number of patients in subgroup; n, number of patients in specific category; NSCLC, non-small cell lung cancer; PD-L1, Programmed Cell Death Ligand 1.

Table S5: Demographic and clinical characteristics for patients with KRAS G12C-mutant NSCLC by PD-L1 expression - CGDB

	**PD-L1 < 50%**<br>**N=151**	**PD-L1 ≥ 50%**<br>**N=125**	**P-value**
Age at 1L, n (%)			0.5377
<55 years	7 (4.6)	6 (4.8)	
55 – 64 years	34 (22.5)	33 (26.4)	
65 – 74 years	61 (40.4)	40 (32.0)	
≥ 75 years	49 (32.5)	46 (36.8)	
Gender, n (%)			0.2317
Female	94 (62.3)	68 (54.4)	
Race, n (%)			0.1664
White	108 (71.5)	83 (66.4)	
Black	10 (6.6)	6 (4.8)	
Asian/Other	16 (11.3)	23 (18.4)	
Unknown	16 (10.6)	13 (10.4)	
Histology, n (%)			0.0290
Non-squamous cell carcinoma	143 (94.7)	106 (84.8)	
NSCLC histology NOS	3 (2.0)	7 (5.6)	
Squamous cell carcinoma	5 (3.3)	12 (9.6)	
Smoking Status, n (%)			0.2540
History of smoking	148 (98.0)	125 (100.0)	
No history of smoking	3 (2.0)	0 (0.0)	
Practice Type, n (%)			0.1414
Community	132 (87.4)	116 (92.8)	
Academic	9 (6.0)	7 (5.6)	
Both	10 (6.6)	2 (1.6)	
Stage at Initial Diagnosis, n (%)			0.1190
Advanced (Stage IIIB-IV)	110 (72.8)	104 (83.2)	
Early stage (Stage I-IIIA)	36 (23.8)	18 (14.4)	
Not Reported	5 (3.3)	3 (2.4)	
Performance Status, n (%)			0.8176
0	40 (26.5)	31 (24.8)	
1	66 (43.7)	61 (48.8)	
≥ 2	29 (19.2)	27 (21.6)	
NA	16 (10.6)	6 (4.8)	
CNS/brain metastasis at 1L, n (%)			0.9443
Yes	37 (24.5)	32 (25.6)	

No	114 (75.5)	93 (74.4)	
TP53 mutation, n (%)			0.0119
Mutant	69 (45.7)	77 (61.6)	
Wild type	82 (54.3)	48 (38.4)	
KEAP1 mutation, n (%)			0.0044
Mutant	40 (26.5)	15 (12.0)	
Wild type	111 (73.5)	110 (88.0)	
STK11 mutation, n (%)			<.0001
Mutant	61 (40.4)	16 (12.8)	
WIld type	90 (59.6)	109 (87.2)	

Abbreviations: 1L, first-line treatment; CGDB, Clinico-Genomic Database; CNS, central nervous system; KRAS, Kirsten rat sarcoma viral oncogene homolog; N, total number of patients in subgroup; n, number of patients in specific category; NA, not applicable; NOS, not otherwise specified; NSCLC, non-small cell lung cancer; PD-L1, Programmed Cell Death Ligand 1.

Table S6: Demographic and clinical characteristics in patients with KRAS G12C-mutant NSCLC and PD-L1 ≥ 50% treated with platinum-based chemotherapy plus pembrolizumab versus pembrolizumab monotherapy (EHR database)

	**Platinum-based chemotherapy + pembrolizumab N=126**	**Pembrolizumab monotherapy N=177**	**P-value**
Age at 1L, n (%)			0.002
< 55 years	6 (4.8)	7 (4.0)	
55 – 64 years	38 (30.2)	35 (19.8)	
65 – 74 years	55 (43.7)	60 (33.9)	
≥ 75 years	27 (21.4)	75 (42.4)	
Gender, n (%)			0.0829
Female	65 (51.6)	109 (61.6)	
Race, n (%)			0.8076
White	93 (73.8)	127 (71.8)	
Black	6 (4.8)	8 (4.5)	
Asian/Other	8 (6.3)	16 (9.0)	
Unknown	19 (15.1)	26 (14.7)	
Histology, n (%)			0.109
Non-squamous cell carcinoma	119 (94.4)	158 (89.3)	
Squamous cell carcinoma	1 (0.8)	9 (5.1)	
NSCLC histology NOS	6 (4.8)	10 (5.7)	
Smoking Status, n (%)			0.1419
History of smoking	126 (100.0)	174 (98.3)	
No history of smoking	0 (0.0)	3 (1.7)	
Practice Type, n (%)			0.4805
Community	109 (86.5)	144 (81.4)	
Academic	14 (11.1)	28 (15.8)	
Both	3 (2.4)	5 (2.8)	
Stage at Initial Diagnosis, n (%)			0.0864
Advanced (Stage IIIB-IV)	112 (88.9)	142 (80.2)	
Early stage (Stage I-IIIA)	12 (9.5)	33 (18.6)	
Not Reported	2 (1.6)	2 (1.1)	
Performance Status, n (%)			0.386
0	34 (27.0)	42 (23.7)	
1	56 (44.4)	75 (42.4)	
≥ 2	24 (19.0)	46 (26.0)	
NA	12 (9.5)	14 (7.9)	
STK11 mutation among tested , n (%)			0.8178

Mutant	5 (6.0)	7 (6.9)	
Wild type	78 (94.0)	95 (93.1)	
Unknown/untested	43	75	
KEAP1 mutation among tested, n (%)			0.9162
Mutant	5 (8.1)	6 (8.6)	
Wild type	57 (91.9)	64 (91.4)	
Untested/unknown	64	107	
TP53 mutation among tested , n (%)			0.9928
Mutant	57 (58.8)	70 (58.8)	
Wild type	40 (41.2)	49 (41.2)	
Unknown/untested	29	58	
Documented sites of metastases , n (%)			
Adrenal metastasis at 1L	24 (19.0)	25 (14.1)	0.2513
Bone metastasis at 1L	56 (44.4)	53 (29.9)	0.0095
Brain metastasis at 1L	41 (32.5)	52 (29.4)	0.5565
Liver metastasis at 1L	22 (17.5)	21 (11.9)	0.1689
Pleural metastasis at 1L	29 (23.0)	32 (18.1)	0.2909
Any Metastases at 1L	102 (81.0)	127 (71.8)	0.0662

Abbreviations: 1L, first-line treatment; EHR, electronic health record-derived; KRAS, Kirsten rat sarcoma viral oncogene homolog; N, total number of patients in subgroup; n, number of patients in specific category; NA, not applicable; NOS, not otherwise specified; NSCLC, non-small cell lung cancer; PD-L1, Programmed Cell Death Ligand 1.

Table S7: Demographic and clinical characteristics by KRAS mutation type in patients with NSCLC treated with 1L platinum-based chemotherapy plus pembrolizumab (EHR Database)

	KRAS G12C Mutation N=571	Other KRAS Mutation N=827	KRAS Wild Type With Driver Mutations N=431	KRAS Wild Type Without Driver Mutations N=2792	p value
Age Categories, years n (%)					0.0262
<55	41 (7.2)	71 (8.6)	48 (11.1)	215 (7.7)	
55 - 64	160 (28.0)	236 (28.5)	114 (26.5)	701 (25.1)	
65 - 74	231 (40.5)	294 (35.6)	142 (32.9)	1081 (38.7)	
≥ 75	139 (24.3)	226 (27.3)	127 (29.5)	795 (28.5)	
Gender, n (%)					<.0001
Female	317 (55.5)	445 (53.8)	233 (54.1)	1114 (39.9)	
Male	254 (44.5)	382 (46.2)	198 (45.9)	1677 (60.1)	
Unknown	0 (0.0)	0 (0.0)	0 (0.0)	1 (0.0)	
Race, n (%)					<.0001
Asian	6 (1.1)	7 (0.8)	22 (5.1)	48 (1.7)	
Black	44 (7.7)	74 (8.9)	37 (8.6)	291 (10.4)	
Other race	40 (7.0)	55 (6.7)	43 (10.0)	206 (7.4)	
White	399 (69.9)	594 (71.8)	258 (59.9)	1885 (67.5)	
Unknown	82 (14.4)	97 (11.7)	71 (16.5)	362 (13.0)	
Histology, n (%)					<.0001
Non-squamous cell carcinoma	543 (95.1)	751 (90.8)	375 (87.0)	1823 (65.3)	
Squamous cell carcinoma	13 (2.3)	44 (5.3)	39 (9.0)	848 (30.4)	
NSCLC histology NOS	15 (2.6)	32 (3.9)	17 (3.9)	121 (4.3)	
Smoking Status, n (%)					<.0001
History of smoking	563 (98.6)	773 (93.5)	312 (72.4)	2560 (91.7)	
No history of smoking	8 (1.4)	54 (6.5)	119 (27.6)	230 (8.2)	
Unknown	0 (0.0)	0 (0.0)	0 (0.0)	2 (0.1)	
Stage at Initial Diagnosis, n(%)					0.0142
Advanced (Stage IIIB-IV)	491 (86.0)	703 (85.0)	390 (90.5)	2356 (84.4)	
Early stage (Stage I-IIIA)	73 (12.8)	117 (14.1)	34 (7.9)	406 (14.5)	
Not Reported	7 (1.2)	7 (0.8)	7 (1.6)	30 (1.1)	

Performance Status score, n (%)					0.932<br>7
0	161 (28.2)	233 (28.2)	130 (30.2)	819 (29.3)	
1	257 (45.0)	375 (45.3)	180 (41.8)	1220 (43.7)	
≥ 2	99 (17.3)	137 (16.6)	67 (15.5)	457 (16.4)	
Unknown	54 (9.5)	82 (9.9)	54 (12.5)	296 (10.6)	
EGFR Status, n (%)					<.000<br>1
Negative	540 (94.6)	764 (92.4)	269 (62.4)	2741 (98.2)	
Positive	5 (0.9)	6 (0.7)	160 (37.1)	0 (0.0)	
Untested/Inconclusive	26 (4.6)	57 (6.9)	2 (0.5)	51 (1.8)	
ALK Status, n (%)					<.000<br>1
Negative	525 (91.9)	761 (92.0)	407 (94.4)	2679 (96)	
Positive	1 (0.2)	0 (0.0)	17 (3.9)	0 (0.0)	
Untested/Inconclusive	45 (7.9)	66 (8.0)	7 (1.6)	113 (4)	
BRAF Status, n (%)					<.000<br>1
Negative	517 (90.5)	720 (87.1)	235 (54.5)	2702 (96.8)	
Positive	16 (2.8)	38 (4.6)	190 (44.1)	0 (0.0)	
Untested/Inconclusive	38 (6.7)	69 (8.3)	6 (1.4)	90 (3.2)	
ROS1 Status, n (%)					<.000<br>1
Negative	513 (89.8)	744 (90)	406 (94.2)	2663 (95.4)	
Positive	0 (0.0)	0 (0.0)	10 (2.3)	0 (0.0)	
Untested/Inconclusive	58 (10.2)	83 (10.0)	15 (3.5)	129 (4.6)	
MET Exon 14 Status, n (%)					<.000<br>1
Negative	495 (86.7)	699 (84.5)	364 (84.5)	2461 (88.1)	
Positive	0 (0.0)	0 (0.0)	39 (9.0)	0 (0.0)	
Untested/Inconclusive	76 (13.3)	128 (15.5)	28 (6.5)	331 (11.9)	
RET Rearrangement Status, n (%)					<.000<br>1
Negative	505 (88.4)	710 (85.9)	398 (92.3)	2543 (91.1)	
Positive	2 (0.4)	0 (0.0)	16 (3.7)	0 (0.0)	
Untested/Inconclusive	64 (11.2)	117 (14.1)	17 (3.9)	249 (8.9)	
NTRK Rearrangement Status, n (%)					<.000<br>1
Negative	434 (76.0)	586 (70.9)	356 (82.6)	2109 (75.5)	
Positive	0 (0.0)	0 (0.0)	4 (0.9)	0 (0.0)	
Untested/Inconclusive	137 (24.0)	241 (29.1)	71 (16.5)	683 (24.5)	
STK11 Status, n (%)					<.000<br>1
Negative	282 (49.4)	410 (49.6)	235 (54.5)	1444 (51.7)	
Positive	88 (15.4)	127 (15.4)	21 (4.9)	226 (8.1)	
Untested/Inconclusive	201 (35.2)	290 (35.1)	175 (40.6)	1122 (40.2)	

KEAP1 Status, n (%)					0.0205
Negative	229 (40.1)	312 (37.7)	190 (44.1)	1131 (40.5)	
Positive	34 (6.0)	48 (5.8)	13 (3.0)	106 (3.8)	
Untested/Inconclusive	308 (53.9)	467 (56.5)	228 (52.9)	1555 (55.7)	
TP53 Status, n (%)					<.0001
Negative	215 (37.7)	300 (36.3)	134 (31.1)	631 (22.6)	
Positive	212 (37.1)	319 (38.6)	209 (48.5)	1487 (53.3)	
Untested/Inconclusive	144 (25.2)	208 (25.2)	88 (20.4)	674 (24.1)	
Brain metastasis at 1L, n (%)					<.0001
No/Unknown	410 (71.8)	604 (73.0)	324 (75.2)	2236 (80.1)	
Yes	161 (28.2)	223 (27.0)	107 (24.8)	556 (19.9)	
PD-L1 Status, n (%)					<.0001
<1%	125 (30.9)	212 (34.7)	122 (35.4)	910 (44.0)	
1 to 49%	154 (38.0)	218 (35.7)	132 (38.3)	763 (36.9)	
≥50%	126 (31.1)	181 (29.6)	91 (26.4)	397 (19.2)	
Untested	166	216	86	722	

Abbreviations: 1L, first-line treatment; ALK, anaplastic lymphoma kinase; BRAF, B-Raf proto-oncogene, serine/threonine kinase; EGFR, epidermal growth factor receptor; EHR, electronic health record-derived; KRAS, Kirsten rat sarcoma viral oncogene homolog; MET, MET proto-oncogene, receptor tyrosine kinase; N, total number of patients in subgroup; n, number of patients in specific category; NOS, not otherwise specified; NSCLC, non-small cell lung cancer; NTRK, neurotrophic tyrosine receptor kinase; PD-L1, Programmed Cell Death Ligand 1; RET, rearranged during transfection; ROS1, ROS proto-oncogene 1, receptor tyrosine kinase.

Table S8: Demographic and clinical characteristics by KRAS mutation type in patients with NSCLC PD-L1 ≥ 50% treated with 1L pembrolizumab monotherapy (EHR database)

	KRAS G12C Mutation N=177	Other KRAS Mutation N=245	KRAS Wild Type With Driver Mutations N=89	KRAS Wild Type Without Driver Mutations N=458	p value
Age Categories, years, n (%)					0.0343
< 55	7 (4.0)	4 (1.6)	2 (2.2)	30 (6.6)	
55 - 64	35 (19.8)	42 (17.1)	11 (12.4)	81 (17.7)	
65 - 74	60 (33.9)	98 (40.0)	28 (31.5)	144 (31.4)	
≥ 75	75 (42.4)	101 (41.2)	48 (53.9)	203 (44.3)	
Gender, n (%)					<.0001
Female	109 (61.6)	130 (53.1)	53 (59.6)	194 (42.4)	
Male	68 (38.4)	115 (46.9)	36 (40.4)	264 (57.6)	
Race, n (%)					0.7291
Black	8 (4.5)	17 (6.9)	8 (9.0)	40 (8.7)	
Asian/Other	16 (9.0)	22 (9.0)	7 (7.9)	43 (9.4)	
White	127 (71.8)	172 (70.2)	66 (74.2)	314 (68.6)	
Unknown	26 (14.7)	34 (13.9)	8 (9.0)	61 (13.3)	
Histology, n (%)					<.0001
Non-squamous cell carcinoma	158 (89.3)	225 (91.8)	77 (86.5)	278 (60.7)	
Squamous cell carcinoma	9 (5.1)	9 (3.7)	10 (11.2)	149 (32.5)	
NSCLC histology NOS	10 (5.7)	11 (4.5)	2 (2.2)	31 (6.8)	
Smoking Status, n (%)					<.0001
History of smoking	174 (98.3)	232 (94.7)	71 (79.8)	421 (91.9)	
No history of smoking	3 (1.7)	13 (5.3)	18 (20.2)	37 (8.1)	
Stage at Initial Diagnosis					0.5885
Advanced (Stage IIIB-IV)	142 (80.2)	193 (78.8)	65 (73.0)	363 (79.3)	
Early stage (I-IIIA)	33 (18.6)	51 (20.8)	22 (24.7)	87 (19.0)	
Not Reported	2 (1.1)	1 (0.4)	2 (2.2)	8 (1.7)	
Performance Status score, n (%)					0.8946
0	42 (23.7)	51 (20.8)	23 (25.8)	95 (20.7)	
1	75 (42.4)	99 (40.4)	40 (44.9)	200 (43.7)	
≥ 2	46 (26.0)	65 (26.5)	19 (21.3)	118 (25.8)	
Unknown	14 (7.9)	30 (12.2)	7 (7.9)	45 (9.8)	
EGFR Status, n (%)					<.0001
Negative	165 (93.2)	223 (91.0)	66 (74.2)	452 (98.7)	
Positive	0 (0.0)	1 (0.4)	23 (25.8)	0 (0.0)	

Untested/Inconclusive	12 (6.8)	21 (8.6)	0 (0.0)	6 (1.3)	
ALK Status, n (%)					0.0002
Negative	166 (93.8)	225 (91.8)	82 (92.1)	441 (96.3)	
Positive	0 (0.0)	0 (0.0)	2 (2.2)	0 (0.0)	
Untested/Inconclusive	11 (6.2)	20 (8.2)	5 (5.6)	17 (3.7)	
BRAF Status, n (%)					<.0001
Negative	155 (87.6)	210 (85.7)	47 (52.8)	447 (97.6)	
Positive	9 (5.1)	10 (4.1)	40 (44.9)	0 (0.0)	
Untested/Inconclusive	13 (7.3)	25 (10.2)	2 (2.2)	11 (2.4)	
ROS1 Status, n (%)					0.1004
Negative	162 (91.5)	222 (90.6)	83 (93.3)	436 (95.2)	
Positive	0 (0.0)	0 (0.0)	0 (0.0)	0 (0.0)	
Untested/Inconclusive	15 (8.5)	23 (9.4)	6 (6.7)	22 (4.8)	
MET Exon 14 Status, n (%)					<.0001
Negative	146 (82.5)	197 (80.4)	54 (60.7)	393 (85.8)	
Positive	0 (0.0)	1 (0.4)	23 (25.8)	0 (0.0)	
Untested/Inconclusive	31 (17.5)	47 (19.2)	12 (13.5)	65 (14.2)	
RET Rearrangement Status, n (%)					<.0001
Negative	150 (84.7)	203 (82.9)	77 (86.5)	409 (89.3)	
Positive	0 (0.0)	0 (0.0)	3 (3.4)	0 (0.0)	
Untested/Inconclusive	27 (15.3)	42 (17.1)	9 (10.1)	49 (10.7)	
NTRK Rearrangement Status, n (%)					0.9018
Negative	127 (71.8)	171 (69.8)	64 (71.9)	332 (72.5)	
Positive	0 (0.0)	0 (0.0)	0 (0.0)	0 (0.0)	
Untested/Inconclusive	50 (28.2)	74 (30.2)	25 (28.1)	126 (27.5)	
STK11 Status, n (%)					0.8213
Negative	95 (53.7)	128 (52.2)	50 (56.2)	223 (48.7)	
Positive	7 (4.0)	10 (4.1)	4 (4.5)	18 (3.9)	
Untested/Inconclusive	75 (42.4)	107 (43.7)	35 (39.3)	217 (47.4)	
KEAP1 Status, n (%)					0.3523
Negative	64 (36.2)	86 (35.1)	36 (40.4)	159 (34.7)	
Positive	6 (3.4)	4 (1.6)	1 (1.1)	21 (4.6)	
Untested/Inconclusive	107 (60.5)	155 (63.3)	52 (58.4)	278 (60.7)	
TP53 Status, n (%)					0.0318
Negative	49 (27.7)	65 (26.5)	27 (30.3)	90 (19.7)	
Positive	70 (39.5)	117 (47.8)	41 (46.1)	240 (52.4)	
Untested/Inconclusive	58 (32.8)	63 (25.7)	21 (23.6)	128 (27.9)	
Brain metastasis at 1L, n (%)					0.0004
No/Unknown	125 (70.6)	167 (68.2)	69 (77.5)	373 (81.4)	
Yes	52 (29.4)	78 (31.8)	20 (22.5)	85 (18.6)	

Abbreviations: 1L, first-line treatment; ALK, anaplastic lymphoma kinase; BRAF, B-Raf proto-oncogene, serine/threonine kinase; EGFR, epidermal growth factor receptor; EHR, electronic health record-derived; KRAS, Kirsten rat sarcoma viral oncogene homolog; MET, MET proto-oncogene, receptor tyrosine kinase; N, total number of patients in subgroup; n, number of patients in specific category; NOS, not otherwise specified; NSCLC, non-small cell lung cancer; NTRK, neurotrophic tyrosine receptor kinase; PD-L1, Programmed Cell Death Ligand 1; RET, rearranged during transfection; ROS1, ROS proto-oncogene 1, receptor tyrosine kinase.

Table S9: Patient characteristics by KRAS mutation in patients treated with 1L platinum-based chemotherapy plus pembrolizumab (CGDB)

	KRAS G12C Mutation N=206	Other KRAS Mutation N=304	KRAS Wild Type N=968	p value
Age Categories, years, n (%)				0.4079
<55	18 (8.7)	17 (5.6)	70 (7.2)	
55 - 64	58 (28.2)	81 (26.6)	234 (24.2)	
65 - 74	72 (35.0)	131 (43.1)	408 (42.2)	
≥ 75	58 (28.2)	75 (24.7)	256 (26.5)	
Gender, n (%)				<0001
Female	122 (59.2)	169 (55.6)	394 (40.7)	
Male	84 (40.8)	135 (44.4)	574 (59.3)	
Race, n(%)				0.035
White	137 (66.5)	218 (71.7)	631 (65.2)	
Black	14 (6.8)	31 (10.2)	89 (9.2)	
Asian	2 (1.0)	1 (0.3)	24 (2.5)	
Hispanic or Latino/ Other Race	30 (14.6)	26 (8.6)	114 (11.8)	
Unknown	23 (11.2)	28 (9.2)	110 (11.4)	
Histology, n(%)				0.0005
Non-squamous cell carcinoma	191 (92.7)	281 (92.4)	632 (65.3)	
Squamous cell carcinoma	9 (4.4)	13 (4.3)	298 (30.8)	
NSCLC histology NOS	6 (2.9)	10 (3.3)	38 (3.9)	
Smoking Status, n (%)				0.0005
History of smoking	204 (99.0)	279 (91.8)	868 (89.7)	
No history of smoking	2 (1.0)	25 (8.2)	99 (10.2)	
Unknown/Not documented	0 (0.0)	0 (0.0)	1 (0.1)	
Stage at Initial Diagnosis, n (%)				0.0165
Advanced (Stage IIIB-IV)	172 (83.5)	244 (80.3)	837 (86.5)	
Early stage (I-IIIA)	29 (14.1)	58 (19.1)	122 (12.6)	
Not Reported	5 (2.4)	2 (0.7)	9 (0.9)	
Performance Status score, n (%)				0.9643
0	63 (30.6)	88 (29.0)	281 (29.0)	
1	87 (42.2)	122 (40.1)	409 (42.3)	
≥ 2	36 (17.5)	59 (19.4)	183 (18.9)	
Unknown	20 (9.7)	35 (11.5)	95 (9.8)	
EGFR Mutation, n (%)				0.0005
No	203 (98.5)	302 (99.3)	915 (94.5)	
Yes	3 (1.5)	2 (0.7)	53 (5.5)	
ALK Rearrangement, n (%)				0.0605

No	206 (100.0)	304 (100.0)	957 (98.9)	
Yes	0 (0.0)	0 (0.0)	11 (1.1)	
BRAF Mutation, n (%)				0.0425
No	201 (97.6)	292 (96.1)	908 (93.8)	
Yes	5 (2.4)	12 (4.0)	60 (6.2)	
ROS1 Rearrangement, n (%)				0.7611
No	206 (100.0)	304 (100.0)	964 (99.6)	
Yes	0 (0.0)	0 (0.0)	4 (0.4)	
MET Exon 14, n (%)				0.0005
No	206 (100.0)	304 (100.0)	940 (97.1)	
Yes	0 (0.0)	0 (0.0)	28 (2.9)	
RET Rearrangement, n (%)				0.1999
No	206 (100.0)	304 (100.0)	960 (99.2)	
Yes	0 (0.0)	0 (0.0)	8 (0.8)	
NTRK Rearrangement, n (%)				-
No	206 (100.0)	304 (100.0)	968 (100.0)	
Yes	0 (0.0)	0 (0.0)	0 (0.0)	
TP53 Mutation, n (%)				<.0001
No	102 (49.5)	150 (49.3)	191 (19.7)	
Yes	104 (50.5)	154 (50.7)	777 (80.3)	
KEAP1 Mutation, n (%)				0.0054
No	159 (77.2)	245 (80.6)	828 (85.5)	
Yes	47 (22.8)	59 (19.4)	140 (14.5)	
STK11 Mutation, n (%)				<.0001
No	132 (64.1)	211 (69.4)	808 (83.5)	
Yes	74 (35.9)	93 (30.6)	160 (16.5)	
CNS/brain metastasis at 1L, n (%)				0.0303
No	148 (71.8)	227 (74.7)	768 (79.3)	
Yes	58 (28.2)	77 (25.3)	200 (20.7)	
PD-L1 Status, n (%)				0.0002
<1%	36 (17.5)	63 (20.7)	266 (27.5)	
1 to 49%	46 (22.3)	75 (24.7)	231 (23.9)	
>=50%	38 (18.5)	66 (21.7)	123 (12.7)	
No Evidence of testing	86 (41.8)	100 (32.9)	348 (36.0)	

Abbreviations: 1L, first-line treatment; ALK, anaplastic lymphoma kinase; BRAF, B-Raf proto-oncogene, serine/threonine kinase; CGDB, Clinico-Genomic Database; CNS, central nervous system; EGFR, epidermal growth factor receptor; KRAS, Kirsten rat sarcoma viral oncogene homolog; MET, MET proto-oncogene, receptor tyrosine kinase; N, total number of patients in subgroup; n, number of patients

in specific category; NOS, not otherwise specified; NSCLC, non-small cell lung cancer; NTRK, neurotrophic tyrosine receptor kinase; PD-L1, Programmed Cell Death Ligand 1; RET, rearranged during transfection; ROS1, ROS proto-oncogene 1, receptor tyrosine kinase.

Table S10: Patient characteristics by KRAS Mutation in patients with PD-L1 ≥ 50% treated with 1L pembrolizumab monotherapy (CGDB)

	KRAS G12C Mutation N=46	Other KRAS Mutation N=75	KRAS Wild Type N=137	P value
Age Categories, years, n (%)				0.8831
<55	2 (4.4)	5 (6.7)	7 (5.1)	
55 - 64	8 (17.4)	15 (20.0)	29 (21.2)	
65 - 74	14 (30.4)	29 (38.7)	46 (33.6)	
≥ 75	22 (47.8)	26 (34.7)	55 (40.2)	
Gender, n (%)				0.1582
Female	29 (63.0)	43 (57.3)	66 (48.2)	
Male	17 (37.0)	32 (42.7)	71 (51.8)	
Race, n (%)				0.2939
White	29 (63.0)	53 (70.7)	96 (70.1)	
Black	<5	7 (9.3)	11 (8.0)	
Asian/Other	11 (23.9)	9 (12.0)	15 (11.0)	
Unknown	4 (8.7)	6 (8.0)	15 (11.0)	
Histology, n (%)				0.0005
Non-squamous cell carcinoma	41 (89.1)	67 (89.3)	93 (67.9)	
Squamous cell carcinoma	0 (0.0)	7 (9.3)	6 (4.4)	
NSCLC histology NOS	5 (10.9)	1 (1.3)	38 (27.7)	
Smoking Status, n (%)				0.1164
History of smoking	46 (100.0)	70 (93.3)	126 (92.0)	
No history of smoking	0 (0.0)	5 (6.7)	11 (8.0)	
Stage at Initial Diagnosis, n (%)				0.0815
Advanced (Stage IIIB-IV)	37 (80.4)	52 (69.3)	109 (79.6)	
Early-stage (I-IIIA)	7 (15.2)	23 (30.7)	26 (19.0)	
Not Reported	2 (4.4)	0 (0.0)	2 (1.5)	
Performance Status score, n (%)				0.002
0	3 (6.5)	26 (34.7)	32 (23.4)	
1	28 (60.9)	24 (32.0)	60 (43.8)	
≥ 2	13 (28.3)	20 (26.7)	31 (22.6)	
Unknown	2 (4.4)	5 (6.7)	14 (10.2)	
EGFR Mutation, n (%)				0.1494
No	44 (95.7)	75 (100.0)	132 (96.4)	
Yes	2 (4.4)	0 (0.0)	5 (3.7)	
ALK Rearrangement, n (%)				1.00
No	46 (100.0)	75 (100.0)	136 (99.3)	
Yes	0 (0.0)	0 (0.0)	1 (0.7)	

BRAF Mutation, n (%)				0.5367
No	45 (97.8)	73 (97.3)	129 (94.2)	
Yes	1 (2.2)	2 (2.7)	8 (5.8)	
ROS1 Rearrangement, n (%)				-
No	46 (100.0)	75 (100.0)	137 (100.0)	
Yes	0 (0.0)	0 (0.0)	0 (0.0)	
MET Exon 14, n (%)				0.0048
No	46 (100.0)	75 (100.0)	126 (92.0)	
Yes	0 (0.0)	0 (0.0)	11 (8.0)	
RET Fusion, n (%)				0.6901
No	46 (100.0)	75 (100.0)	135 (98.5)	
Yes	0 (0.0)	0 (0.0)	2 (1.5)	
NTRK Fusion, n (%)				-
No	46 (100.0)	75 (100.0)	137 (100.0)	
Yes	0 (0.0)	0 (0.0)	0 (0.0)	
TP53 Mutation, n (%)				<.0001
No	20 (43.5)	27 (36.0)	18 (13.1)	
Yes	26 (56.5)	48 (64.0)	119 (86.9)	
KEAP1 Mutation, n (%)				0.017
No	42 (91.3)	70 (93.3)	110 (80.3)	
Yes	4 (8.7)	5 (6.7)	27 (19.7)	
STK11 Mutation, n (%)				0.6351
No	41 (89.1)	67 (89.3)	127 (92.7)	
Yes	5 (10.9)	8 (10.7)	10 (7.3)	
CNS/brain metastasis at 1L, n (%)				0.4893
No	37 (80.4)	53 (70.7)	101 (73.7)	
Yes	9 (19.6)	22 (29.3)	36 (26.3)	

Abbreviations: 1L, first-line treatment; ALK, anaplastic lymphoma kinase; BRAF, B-Raf proto-oncogene, serine/threonine kinase; CGDB, Clinico-Genomic Database; CNS, central nervous system; EGFR, epidermal growth factor receptor; KRAS, Kirsten rat sarcoma viral oncogene homolog; MET, MET proto-oncogene, receptor tyrosine kinase; N, total number of patients in subgroup; n, number of patients in specific category; NOS, not otherwise specified; NSCLC, non-small cell lung cancer; NTRK, neurotrophic tyrosine receptor kinase; PD-L1, Programmed Cell Death Ligand 1; RET, rearranged during transfection; ROS1, ROS proto-oncogene 1, receptor tyrosine kinase.
